# Epilepsy, hippocampal sclerosis and febrile seizures linked by common genetic variation around *SCN1A*

**DOI:** 10.1093/brain/awt233

**Published:** 2013-09-06

**Authors:** Dalia Kasperavičiūtė, Claudia B. Catarino, Mar Matarin, Costin Leu, Jan Novy, Anna Tostevin, Bárbara Leal, Ellen V. S. Hessel, Kerstin Hallmann, Michael S. Hildebrand, Hans-Henrik M. Dahl, Mina Ryten, Daniah Trabzuni, Adaikalavan Ramasamy, Saud Alhusaini, Colin P. Doherty, Thomas Dorn, Jörg Hansen, Günter Krämer, Bernhard J. Steinhoff, Dominik Zumsteg, Susan Duncan, Reetta K. Kälviäinen, Kai J. Eriksson, Anne-Mari Kantanen, Massimo Pandolfo, Ursula Gruber-Sedlmayr, Kurt Schlachter, Eva M. Reinthaler, Elisabeth Stogmann, Fritz Zimprich, Emilie Théâtre, Colin Smith, Terence J. O’Brien, K. Meng Tan, Slave Petrovski, Angela Robbiano, Roberta Paravidino, Federico Zara, Pasquale Striano, Michael R. Sperling, Russell J. Buono, Hakon Hakonarson, João Chaves, Paulo P. Costa, Berta M. Silva, António M. da Silva, Pierre N. E. de Graan, Bobby P. C. Koeleman, Albert Becker, Susanne Schoch, Marec von Lehe, Philipp S. Reif, Felix Rosenow, Felicitas Becker, Yvonne Weber, Holger Lerche, Karl Rössler, Michael Buchfelder, Hajo M. Hamer, Katja Kobow, Roland Coras, Ingmar Blumcke, Ingrid E. Scheffer, Samuel F. Berkovic, Michael E. Weale, UK Brain Expression Consortium, Norman Delanty, Chantal Depondt, Gianpiero L. Cavalleri, Wolfram S. Kunz, Sanjay M. Sisodiya

**Affiliations:** 1 NIHR University College London Hospitals Biomedical Research Centre, Department of Clinical and Experimental Epilepsy, UCL Institute of Neurology, Queen Square, London, WC1N 3BG, UK; 2 Epilepsy Society, Chalfont-St-Peter, SL9 0RJ, UK; 3 Immunogenetics Laboratory, University of Porto, 4050-313 Porto, Portugal; 4 UMIB - Instituto Ciências Biomédicas Abel Salazar, University of Porto, 4099-003 Porto, Portugal; 5 Rudolf Magnus Institute of Neuroscience, Department of Neuroscience and Pharmacology, University Medical Centre Utrecht, 3584 CG Utrecht, The Netherlands; 6 Department of Epileptology, University of Bonn, 53105 Bonn, Germany; 7 Life & Brain Centre, University of Bonn, 53105 Bonn, Germany; 8 Epilepsy Research Centre, Austin Health, University of Melbourne, Melbourne VIC 3084, Australia; 9 Department of Molecular Neuroscience, UCL Institute of Neurology, London, WC1N 3BG, UK; 10 Reta Lila Weston Institute, UCL Institute of Neurology, London, WC1N 3BG, UK; 11 Department of Genetics, King Faisal Specialist Hospital and Research Centre, Riyadh, 11211, Saudi Arabia; 12 Department of Medical and Molecular Genetics, King’s College London, Guy's Hospital, London, SE1 9RT, UK; 13 Molecular and Cellular Therapeutics Department, Royal College of Surgeons in Ireland, Dublin 2, Ireland; 14 Brain Morphometry Laboratory, Neurophysics Department, Beaumont Hospital, Dublin 9, Ireland; 15 Department of Neurology, St James’ Hospital, Dublin 8, Ireland; 16 Swiss Epilepsy Centre, 8008 Zurich, Switzerland; 17 Kork Epilepsy Centre, 77694 Kehl-Kork, Germany; 18 Department of Neurology, University Hospital Zurich, 8091 Zurich, Switzerland; 19 Edinburgh and South East Scotland Epilepsy Service, Western General Hospital Edinburgh, EH4 2XU, Scotland, UK; 20 Kuopio Epilepsy Centre, Kuopio University Hospital, 70211 Kuopio, Finland; 21 Department of Neurology, Institute of Clinical Medicine, University of Eastern Finland, 70211 Kuopio, Finland; 22 Paediatric Neurology Unit, Tampere University Hospital and Paediatric Research Centre, University of Tampere, 33521 Tampere, Finland; 23 Department of Neurology, Hôpital Erasme, Université Libre de Bruxelles, 1070 Brussels, Belgium; 24 Department of Paediatrics, Medical University of Graz, 8036 Graz, Austria; 25 Department of Paediatrics, LKH Bregenz, 6900 Bregenz, Austria; 26 Department of Clinical Neurology, Medical University of Vienna, 1090 Vienna, Austria; 27 Groupe Interdisciplinaire de Génoprotéomique Appliquée (GIGA-R) and Faculty of Veterinary Medicine, University of Liège, 4000 Liège, Belgium; 28 Unit of Gastroenterology, Centre Hospitalier Universitaire, University of Liège, 4000 Liège, Belgium; 29 Department of Neuropathology, MRC Sudden Death Brain Bank Project, University of Edinburgh, Wilkie Building, Edinburgh, EH8 9AG, UK; 30 Departments of Medicine and Neurology, Royal Melbourne Hospital, University of Melbourne, Melbourne VIC 3050, Australia; 31 Melbourne Brain Centre, University of Melbourne, Melbourne VIC 3084, Australia; 32 Department of Medicine, Austin Health, University of Melbourne, Melbourne VIC 3084, Australia; 33 Department of Neurosciences, Laboratory of Neurogenetics, University of Genoa, ‘G. Gaslini’ Institute, 16147 Genova, Italy; 34 Paediatric Neurology and Muscular Diseases Unit, Department of Neurosciences, Rehabilitation, Ophthalmology, Genetics, Maternal and Child Health, University of Genoa, ‘G. Gaslini’ Institute, 16147 Genova, Italy; 35 Department of Neurology, Thomas Jefferson University, Philadelphia, PA 19107, USA; 36 Department of Biomedical Science, Cooper Medical School of Rowan University, Camden, NJ 08103, USA; 37 Centre for Applied Genomics, The Children’s Hospital of Philadelphia, Perelman School of Medicine, University of Pennsylvania, Philadelphia, PA 19104-4318, USA; 38 Department of Neurological Disorders and Senses, Hospital Santo António / Centro Hospitalar do Porto, 4099-001 Porto, Portugal; 39 Instituto Nacional de Saúde Dr. Ricardo Jorge (INSA), 4049-019 Porto, Portugal; 40 Department of Medical Genetics, University Medical Centre Utrecht, 3584 CG Utrecht, The Netherlands; 41 Department of Neuropathology, University of Bonn, 53105 Bonn, Germany; 42 Department of Neurosurgery, University of Bochum, 44892 Bochum, Germany; 43 Epilepsy-Centre Hessen, Department of Neurology, University Hospitals and Philipps-University Marburg, 35043 Marburg, Germany; 44 Department of Neurology and Epileptology, Hertie Institute for Clinical Brain Research, University of Tübingen, 72076 Tübingen, Germany; 45 Department of Neurosurgery, University Hospital Erlangen, 91054 Erlangen, Germany; 46 Department of Neurology, Epilepsy Centre, University Hospital Erlangen, 91054 Erlangen, Germany; 47 Department of Neuropathology, University Hospital Erlangen, 91054 Erlangen, Germany; 48 Florey Institute of Neuroscience and Mental Health, Melbourne VIC 3010, Australia; 49 Department of Paediatrics, University of Melbourne, Royal Children’s Hospital, Melbourne VIC 3052, Australia; 50 Department of Neurology, Beaumont Hospital, Dublin 9, Ireland

**Keywords:** mesial temporal lobe epilepsy, mesial temporal sclerosis, *SCN1A*, association, complex genetics

## Abstract

Epilepsy comprises several syndromes, amongst the most common being mesial temporal lobe epilepsy with hippocampal sclerosis. Seizures in mesial temporal lobe epilepsy with hippocampal sclerosis are typically drug-resistant, and mesial temporal lobe epilepsy with hippocampal sclerosis is frequently associated with important co-morbidities, mandating the search for better understanding and treatment. The cause of mesial temporal lobe epilepsy with hippocampal sclerosis is unknown, but there is an association with childhood febrile seizures. Several rarer epilepsies featuring febrile seizures are caused by mutations in *SCN1A*, which encodes a brain-expressed sodium channel subunit targeted by many anti-epileptic drugs. We undertook a genome-wide association study in 1018 people with mesial temporal lobe epilepsy with hippocampal sclerosis and 7552 control subjects, with validation in an independent sample set comprising 959 people with mesial temporal lobe epilepsy with hippocampal sclerosis and 3591 control subjects. To dissect out variants related to a history of febrile seizures, we tested cases with mesial temporal lobe epilepsy with hippocampal sclerosis with (overall *n* = 757) and without (overall *n* = 803) a history of febrile seizures. Meta-analysis revealed a genome-wide significant association for mesial temporal lobe epilepsy with hippocampal sclerosis with febrile seizures at the sodium channel gene cluster on chromosome 2q24.3 [rs7587026, within an intron of the *SCN1A* gene, *P* = 3.36 × 10^−9^, odds ratio (A) = 1.42, 95% confidence interval: 1.26–1.59]. In a cohort of 172 individuals with febrile seizures, who did not develop epilepsy during prospective follow-up to age 13 years, and 6456 controls, no association was found for rs7587026 and febrile seizures. These findings suggest *SCN1A* involvement in a common epilepsy syndrome, give new direction to biological understanding of mesial temporal lobe epilepsy with hippocampal sclerosis with febrile seizures, and open avenues for investigation of prognostic factors and possible prevention of epilepsy in some children with febrile seizures.

## Introduction

Mesial temporal lobe epilepsy with hippocampal sclerosis (MTLEHS) is typically a serious epilepsy syndrome and the most common drug-resistant epilepsy (Berg *et al.*, 2010). It is associated with burdensome co-morbidities, such as memory and psychiatric disorders. MTLEHS is the epilepsy most considered for therapeutic neurosurgery. Although surgery is a proven therapy, only ∼50% of patients have sustained postoperative seizure freedom (de Tisi *et al.*, 2011), and surgery can have important adverse consequences. Better treatment options, or even prevention, of MTLEHS are therefore needed, but rational therapy for MTLEHS remains elusive because its causes are obscure (O’Dell *et al.*, 2012).

MTLEHS is associated with a history of febrile seizures in childhood (Pittau *et al.*, 2009; O’Dell *et al.*, 2012). About 3% of children have febrile seizures; why only some go on to develop epilepsy, including MTLEHS, is unknown. There are a number of rare, genetically-determined, epilepsy syndromes in which febrile seizures are a prominent feature, such as Dravet syndrome and ‘genetic epilepsy with febrile seizures plus’ (GEFS+) (Oliva *et al.*, 2012). MTLEHS has rarely been described in families with GEFS+ (Abou-Khalil *et al.*, 2001) or familial febrile seizures (Mantegazza *et al.*, 2005) associated with *SCN1A* mutations. In familial mesial temporal lobe epilepsy, some family members may have hippocampal sclerosis (Labate *et al.*, 2011). A cluster of families with mesial temporal lobe epilepsy with hippocampal changes has been described in Brazil (Andrade-Valença *et al.*, 2008). Together, this evidence implies genetic susceptibility to MTLEHS, although its heritability is unknown.

We hypothesized that MTLEHS, or MTLEHS with febrile seizures, as common epilepsy syndromes, might be associated with common genetic variation, and tested this ‘common disease-common variant’ hypothesis in a genetic association study.

## Materials and methods

All aspects of the study were approved by the relevant institutional review board. All participants gave written informed consent.

### Subjects

Patients were recruited during clinical appointments. MTLEHS was defined as in Wieser (2004). The diagnosis was made and/or reviewed by a consultant epileptologist who was part of this study, with access to history and investigation results. Patients with bilateral hippocampal sclerosis or dual pathology were excluded. One thousand and eighteen patients were included in the discovery stage and 959 patients in the replication. The number of patients by country is shown in Table 1, with further details in Supplementary Table 1. A history of presence or absence of febrile seizures was accepted only if contemporary medical records or a parental account was available; otherwise it was considered unknown, and not eligible for analysis. Population-based controls (*n* = 7552) were included in the discovery stage, and 3591 in the replication (Table 1 and Supplementary Table 1).

**Table 1:** Number of individuals included in the study, after removal of population outliers and individuals of non-European ancestry

Population	Patients with MTLEHS	Individuals with a definite history of febrile seizures	Individuals with a definite history of no febrile seizures	Controls
**Discovery**				
Austria	157	45	104	332
Belgium	67	23	20	285
USA	71	23	45	605
Finland	116	18	0*	746
Ireland	148	54	90	209
UK	277	117	101	5116
Switzerland	182	61	0*	259
**Total discovery**	**1018**	**341**	**360**	**7552**
**Replication**				
Austria	57	18	39	254
Germany	273	112	161	346
Portugal	102	54	48	190
UK	80	42	28	857
Netherlands	164	74	0*	601
Italy	44	18	26	249
Australia	162	83	79	794
USA	77	15	62	300
**Total replication**	**959**	**416**	**443**	**3591**
**Febrile seizures study**				
Austria	NA	158	NA	585**
Germany	NA	212	NA	346***
UK (ALSPAC)	NA	172	NA	6456
**Total febrile seizures**	NA	**542**	NA	**7387**

NA = not applicable.

*Data were not collected according to the criteria used in the study.

**Combined discovery and replication Austrian controls.

***Same as replication German controls.

We also studied 542 individuals who had had febrile seizures but by the last follow-up had not had unprovoked seizures. These came from three groups: a German group; an Austrian group and the ALSPAC (Avon Longitudinal Study of Parents and Children) cohort, the latter followed to age 13 years (Supplementary material); MTLEHS after febrile seizures almost always develops by the age of 15 (Neligan *et al.*, 2012). These cases were compared with 7387 control subjects from three relevant populations (Table 1). For the German and Austrian samples, the same controls as in the MTLEHS study were used.

To minimize population stratification, only individuals of white European ancestry were included. In the discovery stage, a combination of self-identified ancestry and EIGENSTRAT principal component methods was used to determine European ancestry. In the replication and febrile seizures analyses, only self-reported white individuals of European ancestry were included. More detailed ancestry data were available from all sources except Austria, allowing exclusion of individuals self-reported as coming from countries other than those where they were recruited.

### Genotyping and quality control

In the discovery stage, all but the Austrian samples and Belgian controls comprised a subset of a previously described data set (Kasperaviciūte *et al.*, 2010), genotyped on Illumina genome-wide genotyping chips, mostly on Illumina Human610-Quadv1/Human1-2M-DuoCustom. One hundred and fifty-seven Austrian patients and 332 controls were genotyped on Illumina HumanCNV370duo, and 285 Belgian controls were genotyped on Illumina HumanHap300 genotyping chips. Gender and relatedness checks were performed on all samples. The cluster plots of the top-associated single nucleotide polymorphisms were inspected manually. Details are given in Kasperaviciūte *et al.* (2010) and in the online Supplementary material. For replication analysis, several methods were used for genotyping.

### Statistical analysis

In the discovery stage, genome-wide association analysis was performed using PLINK. Only single nucleotide polymorphisms present on both Illumina Human610-Quadv1 and Human1-2M-DuoCustom were analysed. In the discovery stage, we performed logistic regression using an additive model, including all significant EIGENSTRAT axes (assessed using the Tracy-Widom statistic with *P* < 0.05) as covariates. Only single nucleotide polymorphisms with minor allele frequency of ≥ 1% were analysed. Since the replication samples did not have genome-wide data available to calculate EIGENSTRAT axes, we performed stratified analysis using the Cochran-Mantel-Haenszel test for 2 × 2 × 8 stratified case-control subsamples deriving from eight different recruitment countries and self-identified ancestry, using R. The Woolf test was used to assess effect heterogeneity. Meta-analysis of discovery and replication studies was performed using the inverse variance-weighted fixed-effects model as implemented in the GWAMA software (Mägi and Morris*,* 2010). We considered an association to be genome-wide significant at *P* < 5 × 10^−8^.

To fine map the association signal in the discovery stage, we imputed single nucleotide polymorphisms in the 10 Mb region surrounding rs7587026. Imputation was performed using MINIMAC (Howie *et al.*, 2012), and 1000 Genomes Project data (1000 Genomes Project Consortium *et al.*, 2010) as the reference data set. Subsequent association analysis was performed using MACH2DAT (Li *et al.*, 2010) using significant EIGENSTRAT axes as covariates.

Power calculations were performed using Genetic Power Calculator (Purcell *et al.*, 2003).

### Expression analysis

We tested association between genotypes of the two top single nucleotide polymorphisms rs7587026 and rs11692675 and *SCN1A* exons and gene expression in the middle temporal cortex (Brodmann areas 20 and 21) from 78 patients with MTLEHS who had undergone surgical resection, compared with 78 neurologically normal individuals from the MRC Sudden Death Brain and Tissue Bank. We specifically chose not to study the hippocampus to avoid confounding due to tissue changes such as cell loss and gliosis. All samples were randomly hybridized to Affymetrix Human Exon 1.0 ST arrays. Differential expression of *SCN1A* transcripts incorporating the ‘neonatal’ or ‘adult’ exon 5 form (5N or 5A exon, respectively), and expression of non-coding exons 1a and 1b (GenBank accession numbers DQ993522 and DQ993523, respectively) (Martin *et al.*, 2007) in the 5’ region of *SCN1A*, were tested by quantitative RT-PCR as they are not covered by the array. Details are provided in the Supplementary material.

Further, we tested whether the associated single nucleotide polymorphisms have an effect on expression or splicing of any genes in the genome in post-mortem tissue of nine brain regions from 134 control individuals (Supplementary material).

## Results

### Genome-wide association analyses

We performed a two-stage study. For discovery, we first investigated genome-wide association between all MTLEHS and 531 164 single nucleotide polymorphisms in 1018 MTLEHS cases and 7552 controls from seven populations of European descent (Table 1 and Supplementary Table 1). Using logistic regression analysis and correcting for population stratification, suggestive association emerged for three single nucleotide polymorphisms in a region of strong linkage disequilibrium on chromosome 2q24.3 encompassing *SCN1A* and other sodium channel genes (Supplementary Fig. 1). The most strongly associated single nucleotide polymorphism, rs11692675, is within intron 3 of the *SCN1A* full-length transcript variant (NM_001202435.1) {*P* = 5.26 × 10^−8^, odds ratio for G allele [OR(G)] 1.31, 95% confidence interval (CI) 1.19–1.44; Table 2}. Two other single nucleotide polymorphisms within *SCN1A* intron 1, had similarly low *P*-values: rs7587026 (r^2 ^= 0.806 with rs11692675 in CEU population based on 1000 Genomes data set), *P* = 1.19 × 10^−7^ [OR(A) = 1.31, 95% CI: 1.19–1.45]; and rs580041 (r^2 ^= 0.806 with rs11692675), *P* = 5.74 × 10^−7^ [OR(A) = 1.29, 95% CI: 1.17–1.43].

**Table 2:** Genotype counts, allele frequencies and association results for rs7587026 and rs11692675 SNPs in the MTLEHS study

SNP	*n* patients	*n* controls	Minor allele	Genotype count in patients	Genotype count in controls	Minor allele frequency in patients	Minor allele frequency in controls	*P*-value*	Odds ratio (95% CI)
**Discovery**									
***MTLEHS versus controls***									
rs7587026	1017	7549	A	99/440/478	536/2895/4118	0.314	0.263	1.19 × 10^–7^	1.31 (1.19–1.45)
rs11692675	1018	7547	G	147/477/394	794/3352/3401	0.379	0.327	5.26 × 10^–8^	1.31 (1.19–1.44)
***MTLEHS+FS versus controls***									
rs7587026	341	7549	A	43/161/137	536/2895/4118	0.362	0.263	2.64 × 10^–8^	1.59 (1.35–1.87)
rs11692675	341	7547	G	61/163/117	794/3352/3401	0.418	0.327	1.25 × 10^–6^	1.49 (1.27–1.75)
***MTLEHS−FS versus controls***									
rs7587026	359	6544	A	30/143/186	469/2528/2547	0.283	0.265	0.21	1.12 (0.94–1.33)
rs11692675	360	6542	G	48/167/145	698/2951/2893	0.365	0.332	0.039	1.19 (1.01–1.40)
***MTLEHS+FS versus MTLEHS−FS***									
rs7587026	341	359	A	43/161/137	30/143/186	0.362	0.283	1.12 × 10^–3^	1.48 (1.17–1.87)
rs11692675	341	360	G	61/163/117	48/167/145	0.418	0.365	0.030	1.28 (1.03–1.59)
**Replication**									
***MTLEHS versus controls***									
rs7587026	933	3537	A	89/360/484	247/1361/1929	0.288	0.262	0.025	1.15 (1.02–1.29)
rs11692675	826	3568	G	108/364/354	394/1615/1559	0.351	0.337	0.19	1.08 (0.96–1.21)
***MTLEHS+FS versus controls***									
rs7587026	406	3537	A	43/163/200	247/1361/1929	0.307	0.262	5.88 × 10^–3^	1.26 (1.07–1.48)
rs11692675	371	3568	G	56/156/159	394/1615/1559	0.361	0.337	0.12	1.14 (0.97–1.34)
***MTLEHS−FS versus controls***									
rs7587026	436	2972	A	42/160/234	216/1136/1620	0.280	0.264	0.20	1.11 (0.95–1.31)
rs11692675	357	2983	G	42/164/151	336/1332/1315	0.347	0.336	0.35	1.08 (0.92–1.27)
***MTLEHS+FS versus MTLEHS−FS***									
rs7587026	338	436	A	35/137/166	42/160/234	0.306	0.280	0.22	1.16 (0.93–1.44)
rs11692675	298	357	G	46/125/127	42/164/151	0.364	0.347	0.50	1.09 (0.87–1.36)
**Combined (meta-analysis)**									
***MTLEHS versus controls***									
rs7587026	1950	11 086	A	188/800/962	783/4256/6047	0.302	0.263	3.78 × 10^–8^	1.24 (1.15–1.34)
rs11692675	1844	11 115	G	255/841/748	1188/4967/4960	0.366	0.330	4.87 × 10^–7^	1.21 (1.12–1.30)
***MTLEHS+FS versus controls***									
rs7587026	747	11 086	A	86/324/337	783/4256/6047	0.332	0.263	3.36 × 10^–9^	1.42 (1.26–1.59)
rs11692675	712	11 115	G	117/319/276	1188/4967/4960	0.388	0.330	4.78 × 10^–6^	1.30 (1.16–1.46)
***MTLEHS−FS versus controls***									
rs7587026	795	9516	A	72/303/420	685/3664/5167	0.281	0.265	0.067	1.12 (0.99–1.25)
rs11692675	717	9525	G	90/331/296	1034/4283/4208	0.356	0.333	0.033	1.13 (1.01–1.27)
***MTLEHS+FS versus MTLEHS−FS***									
rs7587026	679	795	A	78/298/303	72/303/420	0.334	0.281	1.53 × 10^–3^	1.30 (1.10–1.52)
rs11692675	639	717	G	107/288/244	90/331/296	0.393	0.356	0.039	1.18 (1.01–1.38)

*In discovery stage, *P*-value is logistic regression *P*-value for additive genetic model; in replication stage, Cochran-Mantel-Haenszel test *P*-value.

*SCN1A* encodes brain-expressed voltage-gated sodium channel type I, alpha subunit. It bears the largest number of known epilepsy-related mutations, some associated with febrile seizures (Oliva *et al.*, 2012). The common *SCN1A* single nucleotide polymorphism rs3812718, affecting splicing (Heinzen *et al.*, 2007), has also been associated with febrile seizures (Schlachter *et al.*, 2009), though replication has failed (Petrovski *et al.*, 2009). Retrospective studies show association between MTLE and febrile seizures (Pittau *et al.*, 2009; O’Dell *et al.*, 2012). Whether febrile seizures cause MTLEHS (Koyama *et al.*, 2012) or whether pre-existing hippocampal abnormalities predispose to febrile seizures (Cendes*,* 2004), which may then also be injurious, is unknown. Clinical differences between patients with and without a history of febrile seizures suggest MTLEHS is heterogeneous (Thom *et al.*, 2010).

**Figure 1:**
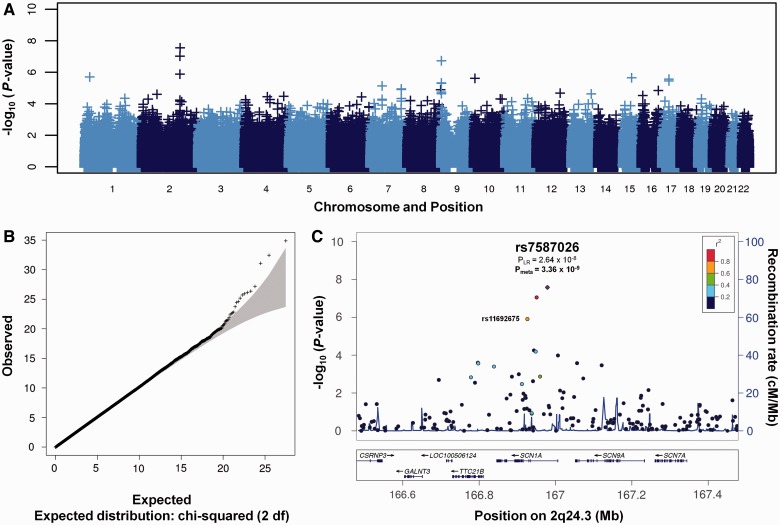
The results of genome-wide association analysis in MTLEHS+FS in discovery stage. (**A**) Manhattan plot, −log_10_ (*P*-values) of the logistic regression test are plotted against single nucleotide polymorphism positions on each chromosome. (**B**) Quantile-quantile plot, the grey shaded area represents the 95% confidence interval of expected −log_10_ (*P*-values). Black dots represent the observed *P*-values; λ = 1.022. (**C**) Regional association results for the chromosome 2q24.3 locus. The left *y*-axis represents −log_10_ (*P*-values) for association with MTLEHS, the right *y*-axis represents the recombination rate, and the *x*-axis represents base-pair positions along the chromosome (human genome Build 37). The top single nucleotide polymorphism, rs7587026, is shown in purple, the rest of the single nucleotide polymorphisms are coloured according to their linkage disequilibrium r^2^ value with rs7587026.

This evidence motivated our pre-analysis collection of febrile seizure data, and our previous study of febrile seizures (Petrovski *et al.*, 2009). We performed analysis of patients in the discovery cohort with a known history of presence of childhood febrile seizures (MTLEHS + FS, *n* = 341) (Table 2 and Fig. 1). The strongest association was for rs7587026, *P* = 2.64 × 10^−8^ [OR(A) = 1.59, 95% CI: 1.35–1.87] and rs580041, *P* = 8.91 × 10^−7^ [OR(A) = 1.56, 95% CI: 1.33–1.84], whereas the signal for rs11692675 was slightly weaker, *P* = 1.25 × 10^−6^ [OR(G) = 1.49, 95% CI: 1.27–1.75]. No association was seen in patients with MTLEHS without febrile seizures (MTLEHS−FS), despite similar sample size.

To refine the association signal, we performed regional imputation in the discovery data set of a 10 Mb region surrounding rs7587026 using the 1000 Genomes reference panel. Two single nucleotide polymorphisms had slightly lower *P*-values in the MTLEHS+FS analysis than the original single nucleotide polymorphisms [rs16851603 (*P* = 2.23 × 10^−8^) and rs3919196 (*P* = 2.26 × 10^−8^)], but neither were significantly stronger than the original associations, and these signals reflected regional linkage disequilibrium structure (Supplementary Fig. 2). No known functional variants in *SCN1A*, nor in other genes in the region, were in high linkage disequilibrium with rs7587026. The association signal is localized within one linkage disequilibrium block that also spans the promoter and 5’ UTR region of *SCN1A* (Supplementary Fig. 2).

### Replication and combined analyses

We selected the two top single nucleotide polymorphisms, rs7587026 and rs11692675, for replication in an independent sample of 959 patients with MTLEHS, of whom 416 had MTLEHS+FS, and 3591 population-matched controls of European descent from eight populations (Table 1 and Supplementary Table 1). We did not study rs580041 because of its perfect linkage disequilibrium with rs7587026 in white Europeans (r^2 ^= 1). We detected an association between rs7587026 and MTLEHS + FS, *P* = 5.88 × 10^−3^ [OR(A) = 1.26, 95% CI: 1.07–1.48; Table 2]; this value remains significant at a revised alpha threshold of 6.3 × 10^−3^ after Bonferroni correction for multiple comparisons in the replication cohort. No association was detected for MTLEHS−FS.

**Figure 2:**
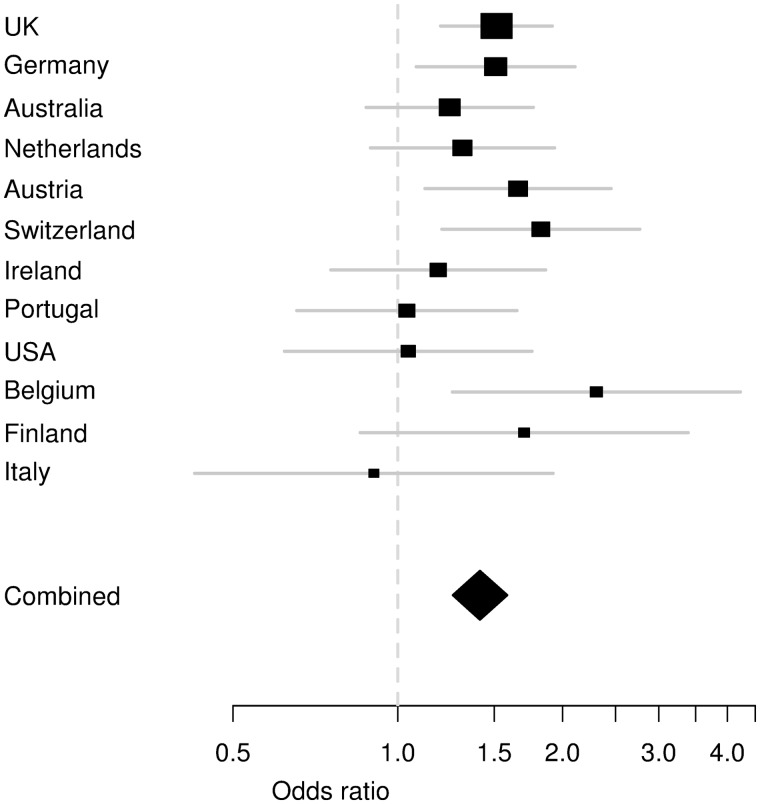
Forest plot for association of rs7587026 with MTLEHS+FS. The confidence interval for each study population is given by a horizontal line, and the point estimate is given by a square whose area is inversely proportional to the standard error of the estimate. The combined odds ratio is drawn as a diamond with horizontal limits at the confidence limits and width inversely proportional to its standard error. The study populations are ordered in descending order by the number of MTLEHS+FS cases.

Meta-analysis of the discovery and replication samples confirmed the association of the 2q24.3 locus with MTLEHS+FS at genome-wide significant level for rs7587026 [*P_meta_* = 3.36 × 10^−9^, OR(A) = 1.42, 95% CI: 1.26–1.59]; the signal for rs11692675 did not reach genome-wide significance [*P_meta_* = 4.78 × 10^−6^, OR(G) = 1.30, 95% CI: 1.16–1.46]. No significant heterogeneity in effect sizes was detected among different populations (Fig. 2, Woolf’s test for heterogeneity *P* = 0.45, see Supplementary Tables 7–10 for allele frequencies in all populations).

### Febrile seizure analysis

To explore whether the observed association with rs7587026 is specific for MTLEHS+FS, or is specific for febrile seizures in general, we examined a total of 7387 controls and three data sets of patients (totalling 542) who had had febrile seizures but had not developed epilepsy by the time of the latest follow-up. It has been shown that almost all children who go on to develop any type of epilepsy after febrile seizures have done so by the age of 15 years (Neligan *et al.*, 2012). Therefore, to address the specificity of the association for MTLEHS+FS rather than febrile seizures alone, the ideal febrile seizures cohort would have been followed to age 15 at least. Of the three data sets available to us, only one met this criterion closely. The ALSPAC prospective cohort, which has the most comprehensive phenotypic data of the three data sets, followed children to age 13: there was no association of rs7587026 with febrile seizures in 171 individuals who did not go on to develop epilepsy (Table 3) in comparison with 6443 controls from the same cohort. The two other cohorts of children with febrile seizures, from Austria [samples partially overlapping with those reported in Schlachter *et al.* (2009)] and Germany, were ascertained at a young age (to six years of age only) and had no follow-up to establish whether the children had febrile seizures only, or febrile seizures in the context of subsequent epilepsy including MTLEHS, and are therefore not best suited to address the question, but were examined as few cohorts overall are available. Bearing this key caveat in mind, in these two data sets there was an observed association of febrile seizures with rs7587026 (Table 3). The previously reported association in the Austrian population with an *SCN1A* functional splice-site single nucleotide polymorphism, rs3812718, was also seen in our Austrian sample [which is not unexpected as there is partial overlap of cases with those in the original report (Schlachter *et al.*, 2009)] and was present in the German sample. The observed association of rs7587026 with febrile seizures disappeared in both Austrian and German data sets when analysis was conditioned on rs3812718 (*P* > 0.19; Table 3). Moreover, although the association of febrile seizures with rs3812718 may be thought to be of interest for pure febrile seizures alone, we note there is no association of rs3812718 with febrile seizures in the best characterized cohort, from ALSPAC (Table 3), nor in a published sample (Petrovski *et al.*, 2009).

**Table 3:** Genotype counts, allele frequencies and association results for rs7587026, rs3812718 and rs922224 in febrile seizures stage

Population	*n* patients	*n* controls	Minor allele	Genotype count in patients	Genotype count in controls	Minor allele frequency in patients	Minor allele frequency in controls	*P*-value in single SNP association (allelic χ^2^ test)	*P*-value in conditional analysis**
**rs7587026**									
Austria	158	584	A	19/58/81	31/216/337	0.304	0.238	0.017	0.19
Germany	194	337	A	15/92/87	20/116/201	0.314	0.231	0.003	0.43
UK (ALSPAC)	171	6443	A	23/59/89	498/2550/3395	0.307	0.275	0.194	0.33*
**rs3812718**									
Austria	133	209	G	16/65/52	52/100/57	0.365	0.488	0.0015	0.030
Germany	212	344	G	32/98/82	88/166/90	0.382	0.497	0.00018	0.0012
**rs922224 (proxy for rs3812718)**									
UK (ALSPAC)	172	6456	G	34/81/57	1371/3144/1941	0.433	0.456	0.40	0.83*

*Conditional analysis performed despite a non-significant single SNP association.

**In conditional analyses, rs7587026 was conditioned for rs3812718 (or its proxy, rs922224, for the ALSPAC cohort), while rs3812718 and rs922224 were conditioned for rs7587026.

Thus, although other single nucleotide polymorphisms in or near *SCN1A* may predispose to pure febrile seizures, the signal we observed in MTLEHS+FS is very unlikely to be due to the history of febrile seizures alone. Moreover, no significant association was detected in a group of patients with other partial epilepsies with a history of febrile seizures [data set from Kasperaviciute *et al.* (2010); rs7587026, *P* = 0.24, OR(A) = 1.15, 95% CI: 0.91–1.45]. The sample for this analysis was smaller (177 patients; 7552 controls), but had 81% power to detect association of OR ≥ 1.42 (as seen in MTLEHS+FS group combined analysis) under 0.05 significance level. Collectively, we found no evidence that the MTLEHS+FS association was due to febrile seizures, or that it holds for all partial epilepsies with febrile seizures.

### *SCN1A* expression in the human brain

The observed association could act by modulating *SCN1A* gene expression. The associated region harbours several alternative untranslated *SCN1A* exons (Martin *et al.*, 2007; Nakayama *et al.*, 2010). We did not detect association between rs7587026 and any protein-coding exon except one (see below) or total *SCN1A* expression, or with expression of untranslated 5’ exons 1a and 1b (Martin *et al.*, 2007) (data not shown) in 78 patients and 78 control subjects.

The presence or absence of transcripts incorporating the ‘neonatal’ *SCN1A* exon 5 (‘5N’) was significantly different according to genotype of the two top single nucleotide polymorphisms (rs11692675 and rs7587026, *P*-values 1.08 × 10^−9^ and 1.17 × 10^−6^, respectively; Supplementary material). For rs11692675 and rs7587026, respectively, none and 1% of the individuals with the GG and AA genotype showed *SCN1A* transcripts in the neonatal form, compared with 83% and 81% with the genotype AA or CC. This alternative splicing event is influenced by rs3812718 (Heinzen *et al.*, 2007). The association of alternative splicing with rs922224 (r^2 ^= 1 with rs3812718) was stronger, *P* = 2.33 × 10^−31^. The level of expression of *SCN1A* exon 5N was also significantly different according to genotype (*P* = 1.62 × 10^−11^ for rs11692675, 2.70 × 10^−6^ for rs7587026, 7.40 × 10^−34^ for rs922224). In conditional analyses including all three single nucleotide polymorphisms, only rs922224 remained significant (*P* = 1.08 × 10^−25^). Finally, expression quantitative trait loci analyses for subsets of patients according to a known history of presence (*n* = 46) or absence (*n* = 27) of febrile seizures in childhood for rs11692675 or rs7587026 showed significant differences in the level of expression of 5N exon according to genotype in both MTLEHS+FS and MTLEHS−FS. Including both rs11692675 or rs11692675 and rs922224 in the regression models, only rs922224 remained significant in both MTLEHS+FS and MTLEHS−FS groups (Supplementary material).

We cannot exclude the possibility that rs7587026 (or another single nucleotide polymorphism in the high linkage disequilibrium region) may act as an additional splicing controller to rs3812718, but our data are consistent with rs7587026 having no solo effect on 5N splicing. We also did not detect any correlation using a significance level of *P* < 5 × 10^−5^ between rs7587026 and expression/splicing of any other genes across the genome (Supplementary material).

## Discussion

We show that common variation in and near *SCN1A* may increase susceptibility to MTLEHS+FS. Our previously published larger genome-wide association study for a broader range of focal epilepsies did not identify any single-single nucleotide polymorphism association (Kasperaviciūte *et al.*, 2010), but the findings here demonstrate that associated variants may exist for more narrowly-defined syndromes. Because the biology of most of the epilepsies is poorly understood, there are few *a priori* data upon which to base selection of the range of phenotypes to include in studies of possible genetic causation. Our findings suggest that focussing on clinically recognized syndromes or constellations (Berg *et al.*, 2010) may prove fruitful by reducing heterogeneity before genomic analyses.

Our association seems to be specific for MTLEHS+FS, with no association for MTLEHS−FS, febrile seizures alone or non-MTLEHS partial epilepsies with febrile seizures. Our findings suggest that there is genetic susceptibility to MTLEHS, and that it, or hippocampal sclerosis, may not necessarily be only acquired. The results support the concept of heterogeneity in MTLEHS, beyond that already documented clinico-pathologically (Tassi *et al.*, 2009; Thom *et al.*, 2010; Blümcke *et al.*, 2012). However, further work will be needed to confirm the specificity of our findings, as we did not formally establish a significant difference in odds ratios between MTLEHS+FS and MTLEHS−FS. It would also be interesting to explore, in a suitably-powered study, whether there is any association with MTLE without hippocampal sclerosis.

The notably weaker association in the replication stage could be due to several factors, the most important of which is the ‘winner’s curse’ (Ioannidis *et al.*, 2009); there may be a large number of weak but real associations in the data, some of which achieve genome-wide significance in a particular study through random stochastic chance, but will not do so in another study. The association in our discovery cohort was replicated in the second independent sample, but it is nevertheless important that other studies are undertaken to further replicate our findings. Other limitations of our study are the lack of genome-wide data in the replication sample, preventing direct population stratification assessment, though self-identification closely corresponds to genetically-determined ancestry (Lao *et al.*, 2008; Wang *et al.*, 2010), a phenomenon we confirmed in the discovery stage, and the small size of some of the replication groups, reducing replication power, and magnifying effects of undetected population admixture.

As for many genome-wide association studies, we could not narrow the association to a single gene or functional variant. There are other genes designated ‘*SCNxA*’ in the vicinity: *SCN3A*, *SCN2A*, *SCN9A* and *SCN7A* (this last does not show any sodium channel activity in exogenous expression systems) (Meisler *et al.*, 2010). Among these genes, *SCN2A* has the most published evidence to support its role in the epilepsies. We cannot exclude the possibility that the association is driven by deleterious variants in these or other nearby genes. *SCN1A*, however, emerges as the most plausible candidate, due both to its proximity to the associated region and its role in other epilepsies with febrile seizures. Notably, our association is with a syndrome involving hippocampal damage, whereas typically no hippocampal damage is observed in patients with Dravet syndrome caused by deleterious changes affecting *SCN1A* (Catarino *et al.*, 2011), suggesting that *SCN1A* might influence epileptogenesis through various mechanisms.

The location of the associated variants within *SCN1A* and overlapping its promoter regions (Long *et al.*, 2008), was suggestive of possible roles in *SCN1A* expression modulation. In fact, we did not detect a definitive effect on expression of *SCN1A* or its exons in temporal neocortex. However, this analysis may have been confounded by many factors: effects may be brain-region or cell-population specific, as in *SCN1A*-related Dravet syndrome, where consequences are only found in interneurons (Ogiwara *et al.*, 2007); our whole-tissue expression analysis would not detect such subtle signals. Moreover, noting the febrile seizures association, the effects may be temporally or spatially restricted, acting only in childhood or/and in the stress of febrile seizures (Koyama *et al.*, 2012). Further studies will be needed to explore possible functional effects.

The detected association could act in different ways, predisposing to MTLEHS+FS as a distinct syndrome, or to the specific development of MTLEHS in the context of remote febrile seizures. If the association does indeed relate to *SCN1A* and function of the encoded protein, new lines of investigation may prove possible in the context of the existing deep knowledge of *SCN1A*, experimental models of MTLE and *in vitro* study of mechanisms of hippocampal dysfunction in epilepsy, as well as intriguing reports of the role of *SCN1A* in many epilepsies, such as the suggestion that mutations in *SCN1A* in Dravet Syndrome may protect against hippocampal sclerosis (Auvin *et al.*, 2008; Catarino *et al.*, 2011). Stratifying by febrile seizures type could also prove illuminating, as prolonged, lateralized or repeated febrile seizures within a short interval may have different effects to ‘uncomplicated’ febrile seizures. Our retrospective febrile seizures data were insufficiently resolved to permit such analysis. This is an important avenue for further investigation, because no predictors exist for the development of epilepsy in the 3% of all the children who have febrile seizures, and because established MTLEHS can have devastating consequences. Eventual reliable prediction of significant risk of MTLEHS after febrile seizures could lead to novel preventative measures in at-risk individuals: here, we note that *SCN1A* encodes an important anti-epileptic drug target and that it is possible to pharmacologically prevent the development of epilepsy after febrile seizures in an animal model (Koyama *et al.*, 2012). Our findings suggest that further work on *SCN1A* variation may contribute to understanding the risk of developing MTLEHS after febrile seizures.

## Supplementary Material

Supplementary Data

## Abbreviations

MTLEHS: mesial temporal lobe epilepsy with hippocampal sclerosis
MTLEHS+FS: MTLEHS with febrile seizures
MTLEHS–FS: MTLEHS without febrile seizures
