# Supplementary material for: Epilepsy, hippocampal sclerosis and febrile seizures linked by common genetic variation around SCN1A
Source: Brain. 2013 Sep 6;136(10):3140–50. doi: 10.1093/brain/awt233 (PMC3784283; doi:10.1093/brain/awt233)
Supplement: Supplementary Data [file supp_136_10_3140__index.html]

Epilepsy, hippocampal sclerosis and febrile seizures linked by common genetic variation around SCN1A — Epilepsy, hippocampal sclerosis and febrile seizures linked by common genetic variation around SCN1A — Supplementary Data 

# Epilepsy, hippocampal sclerosis and febrile seizures linked by common genetic variation around *SCN1A*

## Supplementary Data

files

**Files in this Data Supplement:**

- Supplementary Data - doc file
