# Supplementary material for: Epilepsy, hippocampal sclerosis and febrile seizures linked by common genetic variation around SCN1A
Source: Brain. 2013 Sep 6;136(10):3140–50. doi: 10.1093/brain/awt233 (PMC3784283; doi:10.1093/brain/awt233)
Supplement: Supplementary Data [file supp_awt233_brain-2013-00244-File007.doc]

**Supplementary material**

**Supplementary methods**

1. Patient and control datasets
2. Phenotyping of the ALSPAC cohort
3. Genotyping methods and quality control
   1. Discovery stage
   2. Replication stage
   3. Meta-analysis
4. Expression quantitative trait loci (eQTL) analysis
   1. Expression analysis in MTLEHS patient and control brain tissue
   2. Transcriptome-wide expression analysis in control tissue from 10 brain regions

**Supplementary results**

1. Supplementary Figure 1S. The results of genome-wide association analysis in MTLEHS in discovery stage
2. Supplementary Figure 2S. Regional association results for the chromosome 2q24.3 locus in MTLEHS+FS using directly genotyped and imputed SNPs in discovery stage
3. Supplementary Tables 4S-6S. eQTL analysis results
4. Supplementary Figure 3S. Expression levels (y axes) of neonatal SCN1A exon 5 in patients with MTLEHS and controls by rs11692675 genotypes
5. Supplementary Tables 7S-10S. rs7587026 and rs11692675 genotype counts and allele frequencies in each population in MTLEHS and MTLEHS+FS studies
6. Rare variants in SCN1A

**Supplementary methods**

**1. Patient and controls datasets**

Information on patient and control datasets is provided in the Supplementary Table 1S.

**Supplementary Table 1S.** Patient and control samples used in the study.

| **Population** | **Patient recruitment sites** | **References for patients** | **Controls** | **References for controls** |
| --- | --- | --- | --- | --- |
| **Discovery** | | | | |
| United Kingdom | The National Hospital for Neurology and Neurosurgery, London | A subset of samples from Kasperaviciūt*e et a*l. (2010) | 1958 British Birth Cohort and National Blood Donors, from the Wellcome Trust Case Control Consortium (WTCCC), phase 2 | Wellcome Trust Case Control Consortiu*m et al*., 2007 |
| Belgium | UZ Gasthuisberg, Katholieke Universiteit Leuven, Leuven; and Hôpital Erasme, Université Libre de Bruxelles, Brussels | A subset of samples from Kasperaviciūt*e et a*l. (2010) | Blood donors and healthy volunteers from Belgium | Libioull*e et al*., 2007 |
| USA | Duke University Medical Center, Durham, North Carolina | A subset of samples from Kasperaviciūt*e et a*l. (2010) | Healthy volunteers, 84% of participants filled in a questionnaire about their history of neurological conditions, and the subjects who reported a history of seizures were excluded from the study | Controls from the Duke Memory study (Nee*d et al*., 2009; Cirull*i et al*., 2010); same as in Kasperaviciūt*e et a*l. (2010) |
| Finland | Kuopio University Hospital, Kuopio; Tampere University Hospital, Tampere, Finn-Medi Research Ltd., Tampere; and Research Center of Epilepsy Foundation, Helsinki | A subset of samples from Kasperaviciūt*e et a*l. (2010) | 288 controls from Finland without neurological conditions and 469 population controls from Finland, all 85 years or over old at the time of recruitment (Vantaa85+) | Same as in Kasperaviciūt*e et a*l. (2010); Vantaa85+ described in Myllykanga*s et a*l. (2005) and Peuralinn*a et a*l. (2008) |
| Ireland | Beaumont Hospital, Dublin | A subset of samples from Kasperaviciūt*e et a*l. (2010) | Irish neurologically-normal controls from the Study of Irish Amyotrophic Lateral Sclerosis (SIALS) | Same as in Kasperaviciūt*e et a*l. (2010); SIALS study described in Croni*n et a*l. (2008) |
| Switzerland | University Hospital Zurich; Swiss Epilepsy Center; Epilepsy Center in Kehl/Kork | A subset of samples from Kasperaviciūt*e et a*l. (2010) | controls from Switzerland without neurological conditions | Same as in Kasperaviciūt*e et a*l. (2010) |
| Austria | Department of Clinical Neurology, Medical University of Vienna | Samples partly overlapping with Schlachte*r et a*l. (2009) | Healthy adult volunteers without known diseases | Samples partly overlapping with Schlachte*r et a*l. (2009) |
| **Replication** | | | | |
| Austria | Department of Clinical Neurology, Medical University of Vienna | Samples partly overlapping with Schlachte*r et a*l. (2009) | Healthy adult volunteers without known diseases | Samples partly overlapping with Schlachte*r et a*l. (2009) |
| Germany | University Bonn; Neurological Clinic of the University of Ulm; University Clinic Tübingen; Epilepsy Center Erlangen; and Epilepsy Center Hessen, Philipps-University Marburg | unpublished | Individuals of German descent with a negative life history for neurological or psychiatric disorders based on a personal interview by a psychiatrist with 1 year of training in neurology. | Tod*t et al*., 2006 |
| Portugal | Epilepsy Outpatient Clinic of the Hospital Santo António - Centro Hospitalar do Porto and Immunogenetics Lab – ICBAS – University of Porto | unpublished | Healthy individuals, ethnically and age matched, voluntarily recruited and selected among northern Portuguese blood donors | Part of the control population from Silv*a et a*l. (2009) |
| United Kingdom | The National Hospital for Neurology and Neurosurgery, London | unpublished | Gabriel samples from the 1958 British Birth Cohort, individuals not overlapping with discovery stage. Genotype data has been retrieved from the European Genome-Phenome Archive (EGA, http://www.ebi.ac.uk/ega) which is hosted by EBI, under accession EGAD00000000073 | Moffat*t et al*., 2010Lossin, 2009) |
| Netherlands | University Medical Center Utrecht (UMCU), Utrecht | van der He*l et al*., 2005 | A mixture of random hospital controls and random blood bank donors. All controls were from the Netherlands and of European descent, and at least three of their four grandparents were also born in the Netherlands | Monsuu*r et al*., 2005Lossin, 2009) |
| Italy | Gaslini Institute and collaborative group of Italian League against Epilepsy (LICE) | Mann*a et al*., 2011 | Individuals of Italian descent collected from blood bank donors | unpublished |
| Australia | Epilepsy Program of the Royal Melbourne Hospital, Parkville, Victoria Australia; and the Austin Health Hospital, University of Melbourne, Melbourne, Australia | unpublished | 223 samples from the Australian Blood Bank, and additional 571 non-neurological controls genotyped of which approximately half were Australian women with early-onset breast cancer, and the other half were Australian healthy female controls | Ghoussain*i et al*., 2012 |
| USA | Thomas Jefferson University, Philadelphia | Lohof*f et al*., 2005Lossin, 2009) | Healthy adult volunteers without known diseases | Lohof*f et al*., 2005 |
| **Febrile seizures study** | | | | |
| Austria | Department of Pediatrics, Medical University of Graz; and Department of Pediatrics, LKH Bregenz | Samples partly overlapping with Schlachte*r et a*l. (2009) | Healthy adult volunteers without known diseases | Samples partly overlapping with Schlachte*r et a*l. (2009) |
| Germany | University Bonn | unpublished | Individuals of German descent with a negative life history for neurological or psychiatric disorders based on a personal interview by a psychiatrist with 1 year of training in neurology | Tod*t et al*., 2006Lossin, 2009) |
| United Kingdom | ALSPAC cohort | Boy*d et al.*, 2012 | ALSPAC cohort | Boy*d et al.*, 2012 |

**2. Phenotyping of the ALSPAC cohort**

Criteria used to define pure febrile seizure cases in the ALSPAC study were as follows. Attacks had to be always associated with fever, confirmed by a doctor/at hospital with the diagnosis of febrile seizures made; reaction to immunization as a cause was allowed. Simple or complex febrile seizures were both included. The occurrence of other concomitant clearly non-epileptic episodes (fainting, breath holding) was permissible and did not lead to exclusion from the pure febrile seizure category. Attacks between 6 months and 6 years of age were considered as typical (Sadleir and Scheff*e*r, 2007). If all criteria were met, it was considered to be a pure febrile seizure case. If any single criterion was not clearly met (for instance when not clear if some of the attacks were related to fever), or in presence of atypical features, like attacks occurring before 6 months or after age 6 years, the case was considered as unclear and excluded from analysis. Occurrence of other (including unprovoked) seizure(s) at any point to the age of 13 years (last follow-up) was also an exclusion criterion. Controls were defined as the individuals without history of any seizures until the age of 13 years (last follow-up).

**3. Genotyping methods and quality control**

**3.1 Discovery stage**

In the discovery stage, all but the Austrian samples and Belgian controls comprised a subset of a dataset described previously (Kasperaviciūt*e et al*., 2010), genotyped on Illumina genotyping chips, mostly on Illumina Human610-Quadv1 and Human1-2M-DuoCustom. 157 Austrian patients and 332 controls were genotyped on Illumina HumanCNV370duo chip. The 285 Belgian controls were genotyped on Illumina HumanHap300 v1 and v2 genotyping chips. Gender and relatedness checks were performed on all samples, and the cluster plots of the top associated SNPs were inspected manually.

***Infinium Genotyping Raw Data Analysis***

All patients with epilepsy, except Austrian, and the Switzerland, Finland and USA controls were genotyped at Duke University as a part of genome-wide association study in partial epilepsies (Kasperaviciūt*e et al*., 2010). All samples were processed in batches of 200-500 samples. Samples were clustered using in-house generated Illumina cluster files. After clustering, all samples that had call rates < 98% were deleted. All SNPs that had call frequencies < 100% were then re-clustered. The re-clustering steps may create SNP calling errors, therefore all re-clustered SNPs with HetExcess values between -1.0 to -0.1 and 0.1 to 1.0 and all SNPs with cluster separation values < 0.3 were deleted. Next, to avoid false association resulting from non-random missingness, a "1%" rule was applied: all SNPs for which > 1% of samples were not called, were deleted. These procedures resulted in deletion of 1% to 2.5% of SNPs in different batches and in genotype call rates of 99.93% to 99.96% for the remaining samples and SNPs. 34 duplicate samples were genotyped and the concordance rate for duplicate genotyping was > 99.99%.

***Genotype data processing of WTCCC control samples***

Genotype data has been retrieved from the European Genome-Phenome Archive (EGA, http://www.ebi.ac.uk/ega/) which is hosted by the EBI, under accessions EGAD00000000022 and EGAD00000000024. The genotype data processing was performed as described in Kasperaviciūt*e et a*l. (2010)Lossin, 2009). Shortly, (i) all individuals listed as 'individual exclusions' in the data release documentation were excluded; (ii) any remaining individuals with > 2% missing data were removed; (iii) SNPs with more than 1% missing data were removed; (iv) SNPs with Hardy-Weinberg equilibrium *P*-value below 1 x 10^-10^ were removed; (v) allele frequencies in 1958 Birth cohort and National Blood cohort subsets were compared using the χ^2^ test and SNPs with *P*-values below 1 x 10^-10^ were removed; (vi) principal component analysis was performed on the remaining data using a subset of unlinked SNPs to check for possible plate effects. Such effects were suspected in two plates and these samples were removed.

***Genotype data processing of Irish control samples***

Genotype data has been retrieved from the NINDS Database found at http://www.ncbi.nlm.nih.gov/gap through dbGaP accession number phs000127.v1.p1. The genotype data processing was performed as described in Kasperaviciūt*e et a*l. (2010). SNPs with call rates below 0.98 and cluster separation values below 0.3, as provided in the data release documentation, were removed. We then checked that none of the individuals had > 2% missing data.

***Genotype data processing of Belgian control samples***

The Belgian control dataset was processed as described below: (i) All markers with missing genotypes for more than 4% in the Infinium 1 assay, and with missing genotypes for more that 5% samples in the Infinium 2 assay, were discarded; (ii) All samples with missing genotypes for more than 5% of the markers after step 1, or a mean heterozygosity out of the 31-38% range, or a gender in our records non consistent with the gender predicted from mean homozygosity on X markers (> 98% for males), were discarded; (iii) Additional markers were discarded based on tests for HWE taking only healthy individuals into account (χ^2^ *P*-value threshold of 10^-4^).

***Genotype data processing of Austrian samples***

Any individuals with > 2% missing data were removed and SNPs with more than 1% missing data were removed.

***Gender checks***

The sex of the individuals was imputed using X chromosome data as implemented in PLINK (Purcel*l et al*., 2007). X chromosome homozygosity was estimated for each sample. A male call was assigned if homozygosity exceeded 80%, female if below 20%. Imputed sex was compared with sex in the phenotype database and mismatched samples were removed.

***Relatedness checks***

Identity-by-descent (IBD) was estimated among all pairs of samples as implemented in PLINK, using 65,415 independent SNPs, generated using PLINK option “--indep-pairwise 1500 150 0.2”. Where estimated IBD was > 0.125, one sample from the pair of individuals was removed. If related individuals were concordant for case-control status, the subject with the lower genotyping call rate was removed; if discordant, the control subject was removed from the analysis.

***Manual inspection of top associated SNPs***

The cluster plots of the top associated SNPs were reviewed using Evoker_0.4.3 software (WTCCC data) (<http://en.sourceforge.jp/projects/sfnet_evoker/)> and Beadstudio software (in-house genotyped samples).

***Modified EIGENSTRAT method to control for population stratification***

This standard method was used to correct among gene variants for correlations which arise because of population ancestry rather than disease association (Price *et al.,* 2006). All genotype data were subjected to principal component (PC) analysis. The resulting principal component axes can be used as covariates in subsequent association analysis. We have noticed, however, that some PC axes can be created because of sources of correlations other than population ancestry, such as long linkage disequilibrium (LD) regions or sample processing batch effects. We inspected all EIGENSTRAT axes for these effects and suspect samples were removed.

To correct for LD effects and ensure that EIGENSTRAT axes reflected only effects that applied equally across the whole genome, we (i) excluded known high-LD regions (Supplementary Table 2S) and (ii) thinned the SNP dataset using PLINK option “--indep-pairwise 1500 150 0.2” (such that all SNPs in a window size of 1500 were required to have r^2^ < 0.2), which resulted in a set of 65,415 independent SNPs. This set of SNPs was used in the EIGENSTRAT analysis. Each SNP was regressed on the previous 5 SNPs and the residual entered into the PCA analysis, as suggested by Patterso*n et a*l. (2006). PC axes with *P* < 0.05 as assessed by the Tracy-Widom method Lossin, 2009) were considered statistically significant. To ensure that no axes were dominated by a single high LD region of the genome, we inspected SNP loadings (the "gamma" coefficients of Price e*t a*l. (2006)) for all significant PC axes using Q-Q plots against Normal expectation.

**Supplementary Table 2S.** Known high-LD regions excluded from EIGENSTRAT analysis.

| **Chromosome** | **Start position (NCBI build 36)** | **End position (NCBI build 36)** |
| --- | --- | --- |
| 1 | 48060567 | 52060567 |
| 2 | 85941853 | 100407914 |
| 2 | 134382738 | 137882738 |
| 2 | 182882739 | 189882739 |
| 3 | 47500000 | 50000000 |
| 3 | 83500000 | 87000000 |
| 3 | 89000000 | 97500000 |
| 5 | 44500000 | 50500000 |
| 5 | 98000000 | 100500000 |
| 5 | 129000000 | 132000000 |
| 5 | 135500000 | 138500000 |
| 6 | 25500000 | 33500000 |
| 6 | 57000000 | 64000000 |
| 6 | 140000000 | 142500000 |
| 7 | 55193285 | 66193285 |
| 8 | 8000000 | 12000000 |
| 8 | 43000000 | 50000000 |
| 8 | 112000000 | 115000000 |
| 10 | 37000000 | 43000000 |
| 11 | 46000000 | 57000000 |
| 11 | 87500000 | 90500000 |
| 12 | 33000000 | 40000000 |
| 12 | 109521663 | 112021663 |
| 20 | 32000000 | 34500000 |

**3.2 Replication stage**

The genotyping methods used in replication stage for each population are shown in the Supplementary Table 3S. The primer sequences and reaction conditions can be obtained from the authors. All assays included positive controls for all genotypes and blank controls. 10% of UK patients from the discovery arm were genotyped with the replication samples for quality control: genotype concordance was 100%.

**Supplementary Table 3S.** Genotyping methods in the replication stage.

| **Population** | **Genotyping method** |
| --- | --- |
| Austria | TaqMan allelic discrimination assay (Applied Biosystems) |
| Germany | Restriction fragment length polymorphism assay |
| Portugal | TaqMan allelic discrimination assay (Applied Biosystems) |
| United Kingdom | Patients: TaqMan allelic discrimination assay (Applied Biosystems); Controls: Illumina Human610-Quad chips, publicly available dataset from Gabriel study. |
| Netherlands | TaqMan allelic discrimination assay (Applied Biosystems) |
| Italy | TaqMan allelic discrimination assay (Applied Biosystems) |
| Australia | The MTLEHS and 223 Australian Blood Bank controls were genotyped using the Sequenom™ system by the Australian Genome Research Facility ([www.agrf.com.au](http://www.agrf.com.au/)). The remaining control samples were genotyped using the Illumina Human610-Quad chips |
| USA | Illumina HumanHap550 and Human610-Quad chips. |

**3.3 Meta-analysis**

Meta-analysis of discovery and replication studies was performed using the inverse variance-weighted fixed-effects model as implemented in the GWAMA software (Mägi and Morr*i*s, 2010), where the SNP effect size estimates are weighted by the inverse of the variance of the estimated allelic effect obtained from the corresponding standard error.

**4. Expression and eQTL analyses**

**4.1 Expression analysis in patients with mesial temporal lobe epilepsy and control brain tissue from temporal cortex**

***Brain samples***

Effects of the SNPs identified in the genome-wide association analysis were examined on gene expression in 156 samples: 78 patients and 78 controls (a subset of the controls samples described in section 4.2 below). Surgical specimens were obtained from patients with well-characterized chronic pharmacoresistant MTLEHS who had previously undergone surgical resection at the National Hospital for Neurology and Neurosurgery (NHNN). All brain tissue samples from neurologically healthy control individuals here described were collected from the MRC Sudden Death Brain and Tissue Bank in Edinburgh, UK (Milla*r et al*., 2007). There were no cases of epilepsy or SUDEP in the 78 controls. Informed and written consent was obtained from patients and controls for all procedures as approved by the institutional review board.

Patient and control RNA were isolated from the middle temporal cortex (Brodmann areas 20,21).

***RNA isolation and processing***

Total RNA was isolated from human brain tissues based on the single-step method of RNA isolation (Chomczynski and Sacc*h*i, 1987) using the miRNeasy 96 kit (Qiagen, UK). The quality, purity and concentration of the total RNA were determined by electrophoresis on an Agilent Bioanalyzer (measuring RNA integrity number-RIN) and NanoDrop Spectrophotometer (A260:A280 and A260:A230 ratios). Total RNA samples were processed with the Ambion® WT Expression Kit, Affymetrix GeneChip Whole Transcript Sense Target Labeling Assay, and hybridized to the Affymetrix Exon 1.0 ST Arrays following the manufacturers’ protocols. Hybridized arrays were scanned on an Affymetrix GeneChip® Scanner 3000 7G and visually inspected for hybridization artifacts. Further details regarding RNA isolation, quality control and processing are reported in Trabzun*i et a*l. (2011)Lossin, 2009).

#### *Analysis of Affymetrix Exon Array data*

All samples (patients and controls) were randomly hybridized to Affymetrix Human Exon 1.0 ST Arrays. Expression levels were normalized using the Robust Multi-array Analysis (RMA) algorithm restricted to probe sets containing more than three probes, unique hybridization target and supported by evidence from EntrezGene. Principal component analysis (PCA) was performed to secondarily identify sample outliers. Probe sets containing the SNPs or a SNP in high LD (r^2^ > 0.50) were removed from further analysis. These analyses only suggested a possible association of the SNP rs11692675 with expression of *SCN1A gene* in the patient group (*P* = 0.015), when controlling for batch effects (date of hybridization). RIN, gender and age were not included in the model as they did not have an impact on *SCN1A* gene/exon expression. No significant association was found in the control group, all groups together or when examining the individual exons included in the Affymetrix exon array (*P* > 0.05).

***Quantitative real-time PCR***

*SCN1A* transcript-level expression was further assessed using quantitative real-time PCR (qRT-PCR) with ABI Taqman assays on demand for Hs00897337_mH.

The Affymetrix Human Exon 1.0 ST array does not cover exon 5 splice variants (Tat*e et al*., 2005) or non-coding exons 1a and 1b (GenBank accession nos. DQ993522 and DQ993523, respectively Marti*n et a*l. (2007)) in the 5’ upstream region of *SCN1A*. Expression of these *SCN1A* exons of interest was obtained using qRT-PCR in the same cases and controls. Two housekeeping genes RPLP0 (Hs99999902_m1) and UBC (Hs00824723_m1) were used as endogenous controls.

Samples were analysed using Fluidigm 96 Dynamic (Fluidigm Cat.No.BMK‐M‐96.96) arrays with assays being performed in triplicate. The setup was performed in accordance with the manufacturer’s protocol: 100ng RNA used as input in 20µl reverse transcript reaction. The reverse transcription was performed using the High Capacity cDNA Reverse Transcription Kit (ABI, PN4368813) and the cDNA samples were amplified according to the “Fluidigm® Specific Target Amplification Quick Reference Manual”. In short, the cDNA was amplified using target-specific assay (diluted 1:100) and TaqMan PreAmp Master Mix (2X) (ABI, PN 4391128) in a 14-cycle thermal cycler reaction: 95°C 10min and 14 cycles of 95°C 15sec and 60°C 4min. Amplification was performed using standard conditions: 50°C 2min, 95°C 10min and 40 cycles of 95°C 15sec and 60°C 1min. Data was collected using the Fluidigm Real-Time PCR analysis Software v3.0.2. Relative quantification of mRNA was accomplished by comparative Ct, i.e. 2–ddCt method (Livak and Schmittg*e*n, 2001). For each sample, mRNA expression was normalized with respect to the housekeeping genes RPLP0 and UBC using the Ct method and the relative expression level was calculated by 2^(-(DeltaDelta Ct)).

***Genotyping***

Sixty-five patients with MTLEHS had participated in a published epilepsy genome-wide association study performed by our group (Kasperaviciūt*e et al*., 2010) and therefore genotyping data was already available. Most of the samples (63%) had been genotyped on Illumina HumanHap550 chip and the rest on Human610-Quadv1. Thirteen other cases were genotyped prospectively on the Illumina Infinium Omni1-Quad BeadChip. Details of sample collection and genotyping quality control steps are described above.

The SNP rs11692675 failed on the Illumina Infinium Omni1-Quad BeadChip and the genotypes were imputed in cases and controls genotyped on this chip. For quality control the SNP was genotyped again using TaqMan allelic discrimination assay (Applied Biosystems). Two samples failed for a second time and were not included in the eQTL analyses. Genotype concordance was 100%. The SNP rs922224 also failed on the Illumina Infinium Omni1-Quad BeadChip and genotypes for this SNP were only available for 143 samples.

#### *Expression QTL analysis*

Given that SCN1A transcripts that included the neonatal exon 5 were absent in some genotype groups (e.g. none of the individuals with the GG genotype for the SNP rs11692675 showed *SCN1A* transcripts in the neonatal form), additive logistic regression was used to test for association between SNP genotypes and the presence or absence of neonatal SCN1A exon 5 (5N) transcripts. The dependent variable was designated as 0 or 1, with 1 being specified as the presence of 5N exon and 0 the absence of 5N exon. The additive effect of each SNP genotype was tested by coding the genotypes at each locus as 0, 1 and 2 corresponding to the number of minor alleles. We also examined genotypic effects on expression levels of the SNP rs922224, which is a perfect proxy for rs3812718 (r^2^ = 1 in European populations), a SNP located within an intronic splice donor site, that has been shown to modulate the proportion of transcripts incorporating either the *SCN1A* 5A or 5N exons in adult brain tissue and a cellular minigene expression system (Tat*e et al*., 2005; Heinze*n et al*., 2007). The results are presented in the Supplementary Table 4S. All other tested exons were present in all individuals.

Additive linear regression was used to test for association between SNP genotypes and expression levels at either exon or gene level. The effects of several methodological (hybridization day and RIN) and biological covariates (gender, age) on exon/gene expression were tested for significance and added to the linear or logistic regression model when significant. Normalized square root of expression level was regressed on SNP genotypes. The results are presented in the Supplementary Figure 3S.

Forty-six patients have had febrile seizures (MTLEHS+FS) in childhood, 27 did not have (MTLEHS-FS) and for the reminder, it was unknown. eQTL analyses for these subsets of patients according to a known history of presence and absence of FS showed significant differences in the level of expression of 5N exon according to genotype either in the MTLEHS+FS or MTLEHS-FS group. The level of expression of *SCN1A* exon 5N was also significantly different according to genotype. In conditional analyses including rs11692675, rs7587026 and rs922224, only rs922224 remained significant in either group (Supplementary Tables 5S and 6S).

All *P*-values were corrected for multiple comparisons using the False Discovery Rate (*P* = 0.05). Statistical analyses were performed using Partek’s Genomics Suite v6.6 (Partek Incorporated, USA) and/or R.

**4.2 Transcriptome-wide expression analysis in control brain tissue from brain regions**

#### *Collection and dissection of post-mortem human brain tissue*

Central nervous system (CNS) tissues originating from 134 control individuals were collected by the Medical Research Council (MRC) Sudden Death Brain and Tissue Bank, Edinburgh, UK (Milla*r et al*., 2007), and the Sun Health Research Institute (SHRI), an affiliate of Sun Health Corporation, USA (Beac*h et al*., 2008). Samples originating from the MRC Sudden Death Brain and Tissue Bank were removed from whole brains as fresh tissue and anatomical regions of interest were sampled from brain coronal slices at autopsy and immediately flash frozen. In the case of samples originating from the SHRI, whole brains were removed as fresh tissue at autopsy and brain coronal slices were frozen. Anatomical regions of interest were sampled from brain coronal slices on dry ice.

From each individual we analyzed up to 10 brain regions: cerebellar cortex (CRBL), frontal cortex (FCTX), hippocampus (HIPP), medulla (specifically inferior olivary nucleus, MEDU), occipital cortex (specifically primary visual cortex, OCTX), putamen (PUTM), substantia nigra (SNIG), thalamus (THAL), temporal cortex (TCTX) and intralobular white matter (WHMT).

All individuals were confirmed to be neuropathologically-normal by a consultant neuropathologist using histology performed on sections prepared from paraffin-embedded brain tissue blocks. A detailed description of the samples used in the study, tissue processing and dissection is provided in Trabzun*i et a*l. (2011). All samples had fully informed consent for retrieval and were authorized for ethically- approved scientific investigation (Research Ethics Committee number 10/H0716/3).

***RNA isolation and processing***

This was performed as described above within section 4.1.

#### *Analysis of Affymetrix Exon Array data*

All arrays were pre-processed using Robust Multi-array Average normalisation (RMA) (Irizarr*y et al*., 2003) and log2 transformation in Affymetrix Power Tools v1.14-3. In each case, we also calculated the "detection above background" (DABG) metric. After re-mapping the Affymetrix probe sets onto human genome build 19 (GRCh37) and using Netaffx annotation file Release 31 (HuEx-1_0-st-v2 Probeset Annotations), we restricted analysis to 292,000 probe sets which were annotated to have gene names according to NCBI Reference Sequence build 36 and contained at least three uniquely hybrizing probes that were free of common European (frequency > 1%) SNPs or indels (according to the 1000 Genomes Interim Phase v3, March 2012). Gene-level expression was estimated for 26,000 genes by calculating the Winzorised mean (values below 10% and above 90% excluded before calculating the average) signal of all probe sets corresponding to each gene. The resulting expression data were adjusted for brain bank, gender and batch effects in Partek’s Genomics Suite v6.6 (Partek Incorporated, USA).

#### *DNA extraction, genotyping and imputation*

Genomic DNA was extracted from sub-dissected samples (100–200 mg) of human post-mortem brain tissue using Qiagen’s DNeasy Blood & Tissue Kit (Qiagen, UK). All samples were genotyped on the Illumina Infinium Omni1-Quad BeadChip and on the Immunochip, a custom genotyping array designed for the fine-mapping of auto-immune disorders (International Parkinson’s Disease Genomics Consortium and Wellcome Trust Case Control Consortium 2, 2011; Nall*s et al*., 2011). The BeadChips were scanned using an iScan (Illumina, USA) with an AutoLoader (Illumina, USA). GenomeStudio v.1.8.X (Illumina, USA) was used for analysing the data and generating SNP calls.

After standard quality controls (removal of suspected non-European descent individuals, samples with call rate < 95% and checks on reported sex status, cryptic relatedness, autosomal heterozygosity rate check, monomorphic SNPs or call rate < 95%, no genomic position information or redundant SNPs, *P* < 0.0001 for deviation from HWE, genotyping call rate < 95%, fewer than two heterozygotes present, mismatching alleles in 1000G after allowing for strand mismatch), imputation was performed using MaCH (L*i et al*., 2009, 2010) and minimac (Howi*e et al*., 2012) using the European panel of the 1000 Genomes Project (March 2012: Integrated Phase I haplotype release version 3, based on the 2010-11 data freeze and 2012-03-14 haplotypes). We used the resulting ~5.88 million SNPs and ~577,000 indels with good post-imputation quality (R^2^ > 0.50) and minor allele frequency of at least 5%.

#### *Expression QTL analysis*

The QTL analysis was run for each expression profile (either exon-level or gene-level) against every genetic marker (either SNP or indel) in MatrixEQTL (Shabal*i*n, 2012). Subsequent analyses were conducted in R.

**Supplementary results**

**
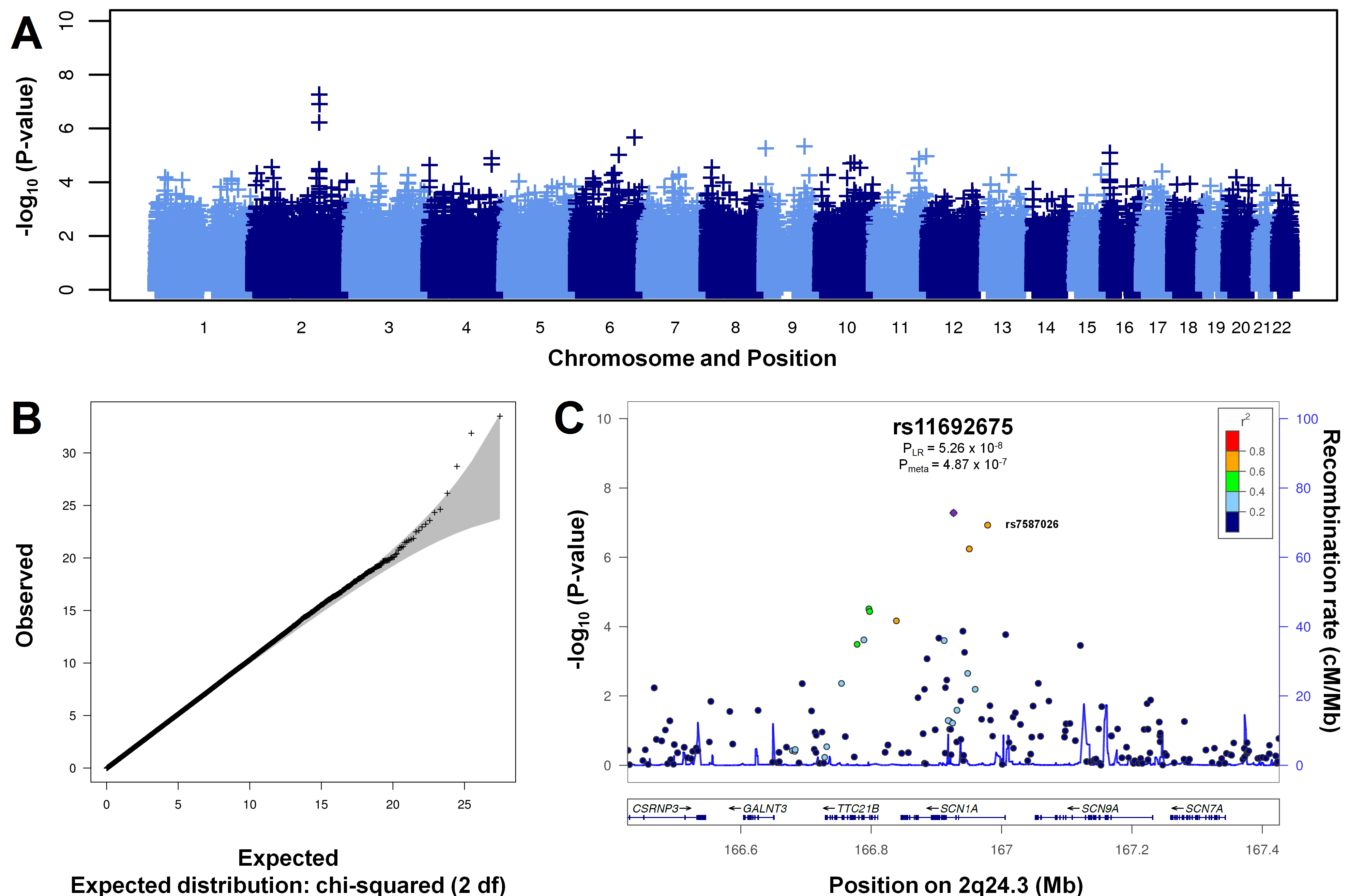
**

**Supplementary Figure 1S.** The results of genome-wide association analysis in MTLEHS in discovery stage. **(A)** Manhattan plot, -log_10_ (*P*-values) of the logistic regression test are plotted against SNP positions on each chromosome; **(B)** Quantile-quantile plot, the gray shaded area represents the 95% confidence interval of expected −log_10_ (*P*-values). Black dots represent the observed *P*-values; λ = 1.029; **(C)** Regional association results for the chromosome 2q24.3 locus. The left *y* axis represents –log_10_ (*P*-values) for association with MTLEHS, the right *y* axis represents the recombination rate, and the *x* axis represents base-pair positions along the chromosome (human genome Build 37). The top SNP, rs11692675 is shown in purple, the rest of SNPs are coloured according to their linkage disequilibrium r^2^ value with rs11692675.

**
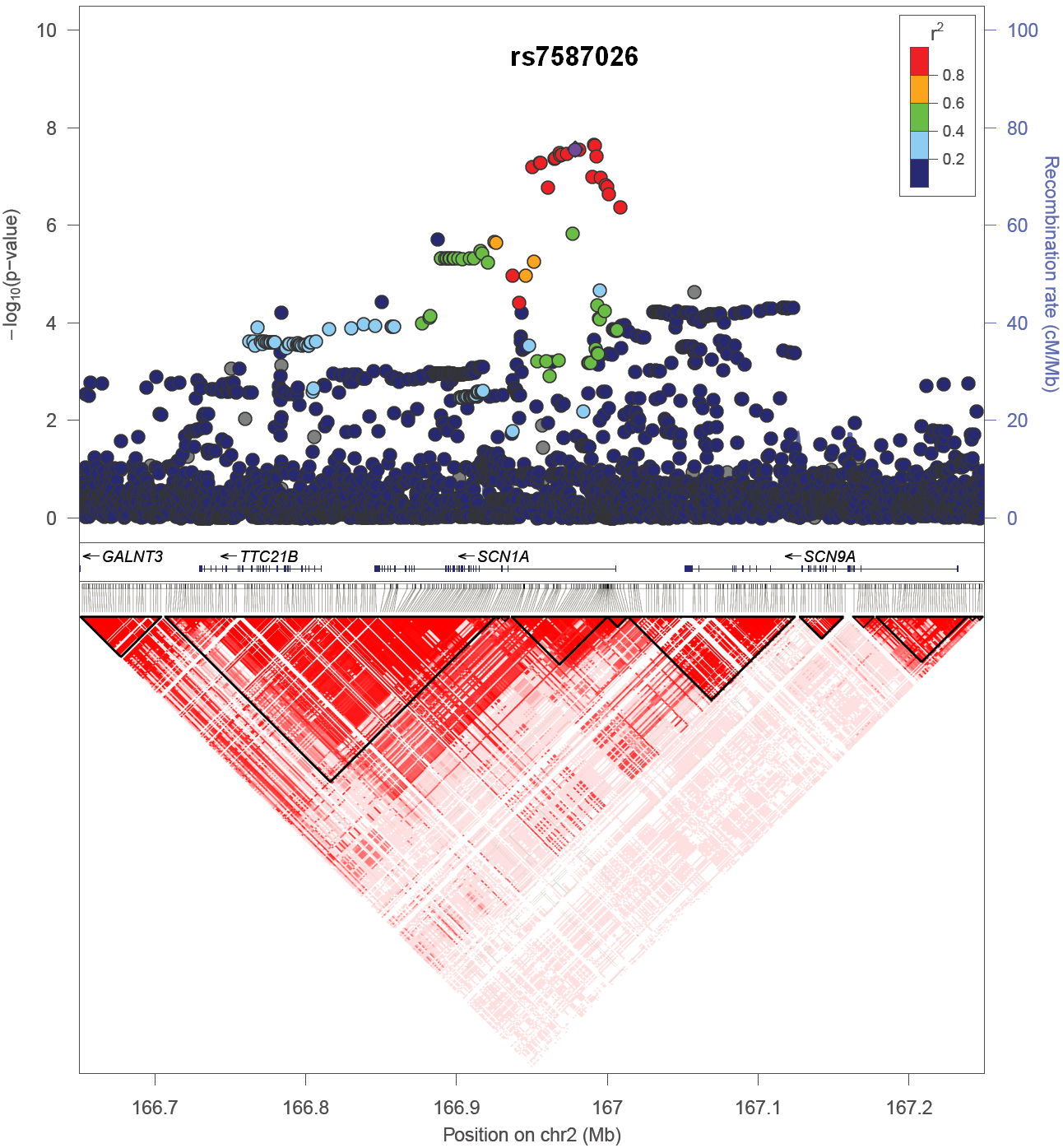
**

**Supplementary Figure 2S.** Regional association results for the chromosome 2q24.3 locus in MTLEHS+FS using directly genotyped and imputed SNPs in the discovery stage. The left y axis represents -log_10_ (*P*-values) for association with MTLEHS, the right y axis represents the recombination rate, and the x axis represents base-pair positions along the chromosome (human genome Build 37). The SNPs are coloured according to their linkage disequilibrium r^2^ value with rs7587026. The colour scheme gradient of the linkage disequilibrium pattern is based on the standard (D' / LOD) option in Haploview software, with red indicating strong (D’ = 1) and significant (LOD > 2) LD. LD-blocks are indicated by black triangles.

**Supplementary Table 4S.** Presence or absence of “neonatal” SCN1A exon 5 form (5N) according to genotype in the SNPs rs11692675, rs7587026 and rs922224.

Cells indicate number of samples. Rows “MTLEHS+5Nexon” indicate number of samples with MTLEHS and expression of transcripts incorporating the *SCN1A* exon 5N form according to genotype; in brackets the total number of samples with MTLEHS and the genotype at the corresponding column. For example no individuals with MTLEHS and genotype GG in the SNP rs11692675 showed expression of the exon 5N. A total of 13 samples with MTLEHS have the genotype GG for the SNP rs11692675.

Rows “controls+5Nexon” indicate number of control samples with expression of transcripts incorporating the *SCN1A* exon 5N form according to genotype; In brackets the total number of controls with the genotype at the corresponding column. For example 24 controls with the genotype AA showed expression of the exon 5N. A total of 29 controls have the genotype AA for the SNP rs11692675.

*P*-value “Cohort” indicates *P*-values for each cohort (patients or controls independently). *P*-value “All” indicates *P*-values for both cohorts together.

| **SNP** | **Genotypes** | | | ***P*-values** | |
| --- | --- | --- | --- | --- | --- |
| **rs11692675** | **GG** | **GA** | **AA** | **Cohort** | **All*** |
| MTLEHS+5Nexon(total MTLEHS) | 0(13) | 25(39) | 21(25) | 6.53 x 10^-7^ | 1.08 x 10^-9^ |
| controls+5Nexon(total controls) | 0(5) | 32(43) | 24(29) | 0.00102 |
| **rs7587026** | **AA** | **AC** | **CC** | **Cohort** | **All*** |
| MTLEHS+5Nexon(total MTLEHS) | 0(7) | 15(34) | 31(37) | 6.29 x 10^-7^ | 1.17 x 10^-6^ |
| controls+5Nexon(total controls) | 1(4) | 24(34) | 31(40) | 0.045 |
| **rs922224** | **CC** | **CT** | **TT** | **Cohort** | **All*** |
| MTLEHS+5Nexon(total MTLEHS) | 9(9) | 31(32) | 0(24) | 1.19 x 10^-18^ | 2.33 x 10^-31^ |
| controls+5Nexon(total controls) | 12(13) | 42(45) | 2(20) | 2.86 x 10^-14^ |

* = in conditional analyses, including both SNPs rs11692675 or rs7587026 and rs922224, only rs922224 remained significant (*P*-value: 2.11 x 10^-24^ and 9.77 x 10^-28^ respectively).

**Supplementary Table 5S**. Presence or absence of “neonatal” SCN1A exon 5 form (5N) according to genotype and presence (+FS) or absence (-FS) of febrile seizures in the SNPs rs11692675, rs7587026 and rs922224.

Cells indicate number of samples. Rows “5Nexon” indicate number of samples with MTLEHS and expression of transcripts incorporating the SCN1A exon 5N form according to genotype; in brackets the total number of samples with MTLEHS and the genotype at the corresponding column.

*P*-value indicates *P*-values for patients with FS (MTLEHS+FS) and without FS (MTLE-FS) in childhood. Conditional *P*-values indicate *P*-values for the logistic regression model including rs11692675 or rs7587026 and rs922224. For example, including both rs11692675 and rs922224 in the regression model, only rs922224 remained significant with a *P*-value of 5.20 x 10^-11^ in the group of patients with MTLEHS+FS. ns = not significant (FDR > 0.05).

| **MTLEHS+FS** | | | | | | |
| --- | --- | --- | --- | --- | --- | --- |
| **SNP** | **Genotypes** | | | ***P*-values** | **Conditional *P*-values** | |
| rs11692675 | GG | GA | AA | 0.0001 |  | ns |
| 5Nexon (totalMTLEHS+FS) | 0(6) | 14(22) | 15(17) |
| rs7587026 | AA | AC | CC | 0.0002 | ns |  |
| 5Nexon (totalMTLEHS+FS) | 0(4) | 9(18) | 21(24) |
| rs922224 | CC | CT | TT | 7.79 x 10^-11^ | 5.20 x 10^-7^ | 1.12 x 10^-7^ |
| 5Nexon (totalMTLEHS+FS) | 6(6) | 21(22) | 0(12) |
| **MTLEHS-FS** | | | | | | |
| **SNP** | **Genotypes** | | | ***P*-values** | **Conditional *P*-values** | |
| rs11692675 | GG | GA | AA | 0.014 |  | ns |
| 5Nexon (totalMTLEHS-FS) | 0(6) | 11(15) | 4(6) |
| rs7587026 | AA | AC | CC | 0.0008 | ns |  |
| 5Nexon (totalMTLEHS-FS) | 0(3) | 6(14) | 9(10) |
| rs922224 | CC | CT | TT | 1.16 x 10^-6^ | 6.18 x 10^-5^ | 2.74 x 10^-7^ |
| 5Nexon (totalMTLEHS-FS) | 3(3) | 9(9) | 0(10) |

**Supplementary Table 6S.** Differences in levels of SCN1A exon 5N expression according to genotype and presence (+FS) or absence (-FS) of febrile seizures.

*P*-value indicates *P*-values for patients with FS (MTLEHS+FS) and without FS (MTLE-FS) in childhood. Conditional *P*-values indicate *P*-values for the linear regression model including the 3 SNPs. ns = not significant.

| **SNP** | ***P*-Values** | | **Conditional *P*-Values** | |
| --- | --- | --- | --- | --- |
|  | **MTLEHS+FS** | **MTLEHS-FS** | **MTLEHS+FS** | **MTLEHS-FS** |
| rs11692675 | 0.00032 | 0.0031 | ns | ns |
| rs7587026 | 0.00034 | 0.0053 | ns | ns |
| rs922224 | 1.61 x 10^-8^ | 1.61 x 10^-8^ | 3.16 x 10^-8^ | 2.23 x 10^-8^ |

**
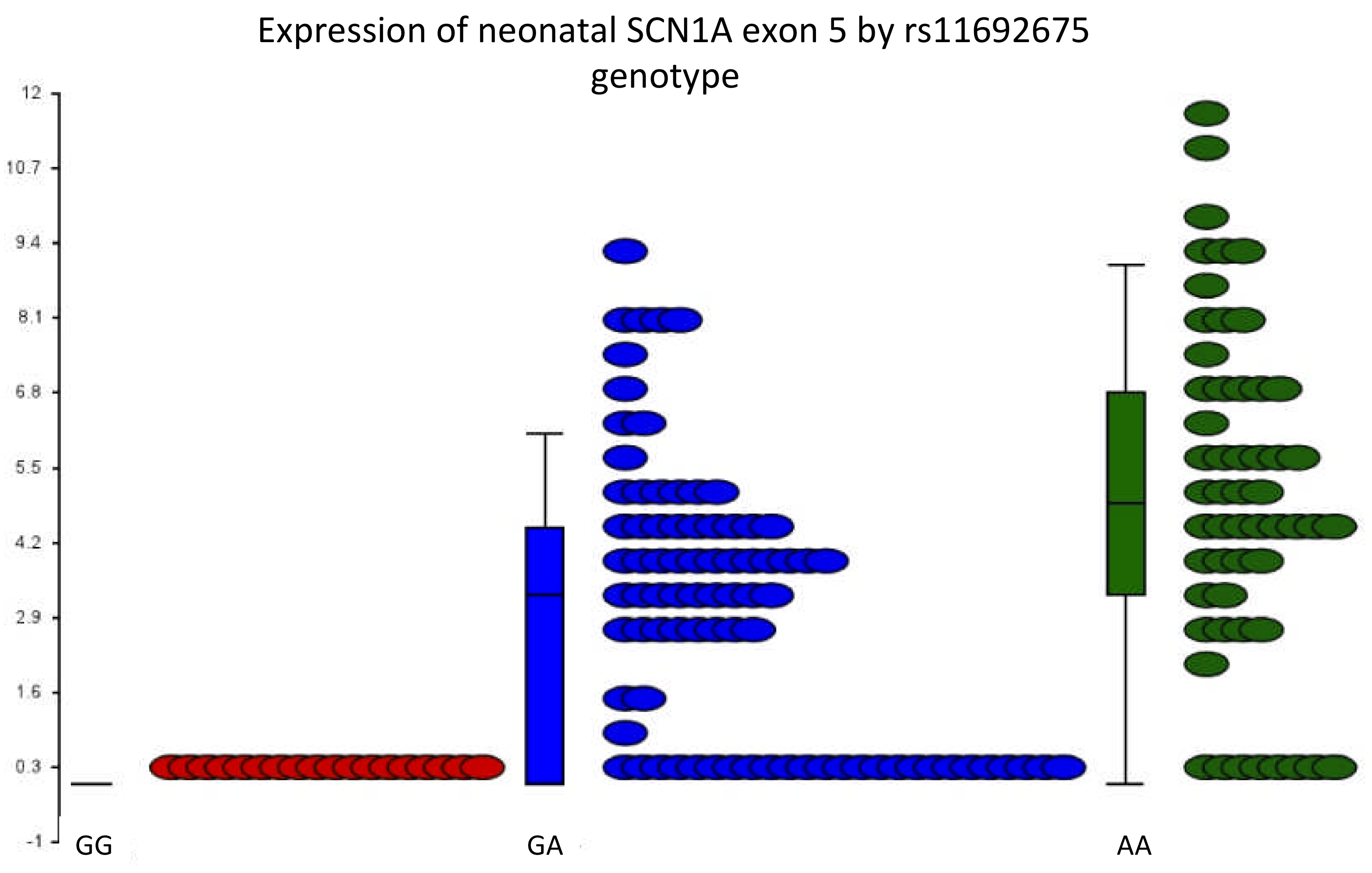
**

**Supplementary Figure 3S**: Expression levels (y axes) of neonatal SCN1A exon 5 in patients with MTLEHS and controls by rs11692675 genotypes. Each dot represents expression level (normalized square root of expression level, y-axis) in an individual. Box plots represent median and 25th–75th percentiles. Upper and lower lines show minimum and maximum values, respectively.

**Supplementary Table 7S.** The genotype counts and allele frequencies of rs7587026 SNP in all populations in the MTLEHS association study. MAF = minor allele frequency.

| **Population** | **Number of patients with MTLEHS** | **Number of controls** | **Genotype counts in patients (AA/AC/CC)** | **Genotype counts in controls (AA/AC/CC)** | **MAF in patients** | **MAF in controls** |
| --- | --- | --- | --- | --- | --- | --- |
| **Discovery** |  |  |  |  |  |  |
| Austria | 156 | 332 | 11/64/81 | 18/121/193 | 0.276 | 0.236 |
| Belgium | 67 | 283 | 11/29/27 | 26/109/148 | 0.381 | 0.284 |
| USA | 71 | 605 | 9/25/37 | 41/225/339 | 0.303 | 0.254 |
| Finland | 116 | 746 | 8/53/55 | 49/274/423 | 0.297 | 0.249 |
| Ireland | 148 | 209 | 15/71/62 | 21/82/106 | 0.341 | 0.297 |
| UK | 277 | 5,115 | 30/110/137 | 363/1,991/2,761 | 0.307 | 0.266 |
| Switzerland | 182 | 259 | 15/88/79 | 18/93/148 | 0.324 | 0.249 |
| **Total discovery** | **1,017** | **7,549** | **99/440/478** | **536/2,895/4,118** | **0.314** | **0.263** |
| **Replication** |  |  |  |  |  |  |
| Austria | 57 | 252 | 4/25/28 | 13/95/144 | 0.289 | 0.240 |
| Germany | 273 | 337 | 28/111/134 | 20/116/201 | 0.306 | 0.231 |
| Portugal | 100 | 184 | 12/41/47 | 19/77/88 | 0.325 | 0.313 |
| UK | 73 | 857 | 9/21/43 | 67/340/450 | 0.267 | 0.277 |
| Netherlands | 150 | 565 | 11/61/78 | 31/225/309 | 0.277 | 0.254 |
| Italy | 41 | 248 | 5/13/23 | 21/106/121 | 0.280 | 0.298 |
| Australia | 162 | 794 | 12/62/88 | 53/294/447 | 0.265 | 0.252 |
| USA | 77 | 300 | 8/26/43 | 23/108/169 | 0.273 | 0.257 |
| **Total replication** | **933** | **3537** | **89/360/484** | **247/1,361/1,929** | **0.288** | **0.262** |
| **Combined** |  |  |  |  |  |  |
| Austria | 213 | 584 | 15/89/109 | 31/216/337 | 0.279 | 0.238 |
| Belgium | 67 | 283 | 11/29/27 | 26/109/148 | 0.381 | 0.284 |
| USA | 148 | 905 | 17/51/80 | 64/333/508 | 0.287 | 0.255 |
| Finland | 116 | 746 | 8/53/55 | 49/274/423 | 0.297 | 0.249 |
| Ireland | 148 | 209 | 15/71/62 | 21/82/106 | 0.341 | 0.297 |
| UK | 350 | 5972 | 39/131/180 | 430/2,331/3,211 | 0.299 | 0.267 |
| Switzerland | 182 | 259 | 15/88/79 | 18/93/148 | 0.324 | 0.249 |
| Germany | 273 | 337 | 28/111/134 | 20/116/201 | 0.306 | 0.231 |
| Netherlands | 150 | 565 | 11/61/78 | 31/225/309 | 0.277 | 0.254 |
| Portugal | 100 | 184 | 12/41/47 | 19/77/88 | 0.325 | 0.313 |
| Italy | 41 | 248 | 5/13/23 | 21/106/121 | 0.280 | 0.298 |
| Australia | 162 | 794 | 12/62/88 | 53/294/447 | 0.265 | 0.252 |
| **Total** | **1,950** | **11,086** | **188/800/962** | **783/4,256/6,047** | **0.302** | **0.263** |

**Supplementary Table 8S.** The genotype counts and allele frequencies of rs7587026 SNP in all populations in the MTLEHS+FS association study. MAF = minor allele frequency.

| **Population** | **Number of patients with MTLEHS+FS** | **Number of controls** | **Genotype counts in patients (AA/AC/CC)** | **Genotype counts in controls (AA/AC/CC)** | **MAF in patients** | **MAF in controls** |
| --- | --- | --- | --- | --- | --- | --- |
| **Discovery** |  |  |  |  |  |  |
| Austria | 45 | 332 | 3/25/17 | 18/121/193 | 0.344 | 0.236 |
| Belgium | 23 | 283 | 5/12/6 | 26/109/148 | 0.478 | 0.284 |
| USA | 23 | 605 | 3/7/13 | 41/225/339 | 0.283 | 0.254 |
| Finland | 18 | 746 | 2/9/7 | 49/274/423 | 0.361 | 0.249 |
| Ireland | 54 | 209 | 6/24/24 | 21/82/106 | 0.333 | 0.297 |
| UK | 117 | 5115 | 17/52/48 | 363/1,991/2,761 | 0.368 | 0.266 |
| Switzerland | 61 | 259 | 7/32/22 | 18/93/148 | 0.377 | 0.249 |
| **Total discovery** | **341** | **7,549** | **43/161/137** | **536/2,895/4,118** | **0.362** | **0.263** |
| **Replication** |  |  |  |  |  |  |
| Austria | 18 | 252 | 1/10/7 | 13/95/144 | 0.333 | 0.240 |
| Germany | 112 | 337 | 9/52/51 | 20/116/201 | 0.313 | 0.231 |
| Portugal | 53 | 184 | 7/20/26 | 19/77/88 | 0.321 | 0.313 |
| UK | 39 | 857 | 6/13/20 | 67/340/450 | 0.321 | 0.277 |
| Netherlands | 68 | 565 | 8/26/34 | 31/225/309 | 0.309 | 0.254 |
| Italy | 18 | 248 | 3/4/11 | 21/106/121 | 0.278 | 0.298 |
| Australia | 83 | 794 | 8/33/42 | 53/294/447 | 0.295 | 0.252 |
| USA | 15 | 300 | 1/5/9 | 23/108/169 | 0.233 | 0.257 |
| **Total replication** | **406** | **3,537** | **43/163/200** | **247/1,361/1,929** | **0.307** | **0.262** |
| **Combined** |  |  |  |  |  |  |
| Austria | 63 | 584 | 4/35/24 | 31/216/337 | 0.341 | 0.238 |
| Belgium | 23 | 283 | 5/12/6 | 26/109/148 | 0.478 | 0.284 |
| USA | 38 | 905 | 4/12/22 | 64/333/508 | 0.263 | 0.255 |
| Finland | 18 | 746 | 2/9/7 | 49/274/423 | 0.361 | 0.249 |
| Ireland | 54 | 209 | 6/24/24 | 21/82/106 | 0.333 | 0.297 |
| UK | 156 | 5,972 | 23/65/68 | 430/2,331/3,211 | 0.356 | 0.267 |
| Switzerland | 61 | 259 | 7/32/22 | 18/93/148 | 0.377 | 0.249 |
| Germany | 112 | 337 | 9/52/51 | 20/116/201 | 0.313 | 0.231 |
| Netherlands | 68 | 565 | 8/26/34 | 31/225/309 | 0.309 | 0.254 |
| Portugal | 53 | 184 | 7/20/26 | 19/77/88 | 0.321 | 0.313 |
| Italy | 18 | 248 | 3/4/11 | 21/106/121 | 0.278 | 0.298 |
| Australia | 83 | 794 | 8/33/42 | 53/294/447 | 0.295 | 0.252 |
| **Total** | **747** | **11,086** | **86/324/337** | **783/4,256/6,047** | **0.332** | **0.263** |

**Supplementary Table 9S.** The genotype counts and allele frequencies of rs11692675 SNP in all populations in the MTLEHS association study. MAF = minor allele frequency.

| **Population** | **Number of patients with MTLEHS** | **Number of controls** | **Genotype counts in patients (GG/GA/AA)** | **Genotype counts in controls (GG/GA/AA)** | **MAF in patients** | **MAF in controls** |
| --- | --- | --- | --- | --- | --- | --- |
| **Discovery** |  |  |  |  |  |  |
| Austria | 157 | 331 | 17/75/65 | 29/125/177 | 0.347 | 0.276 |
| Belgium | 67 | 285 | 11/33/23 | 31/118/136 | 0.410 | 0.316 |
| USA | 71 | 605 | 13/31/27 | 62/260/283 | 0.401 | 0.317 |
| Finland | 116 | 746 | 9/57/50 | 72/288/386 | 0.323 | 0.290 |
| Ireland | 148 | 209 | 20/78/50 | 28/92/89 | 0.399 | 0.354 |
| UK | 277 | 5,112 | 55/113/109 | 548/2,356/2,208 | 0.403 | 0.338 |
| Switzerland | 182 | 259 | 22/90/70 | 24/113/122 | 0.368 | 0.311 |
| **Total discovery** | **1,018** | **7,547** | **147/477/394** | **794/3,352/3,401** | **0.379** | **0.327** |
| **Replication** |  |  |  |  |  |  |
| Austria | 56 | 254 | 4/29/23 | 21/111/122 | 0.330 | 0.301 |
| Germany | 150 | 339 | 21/70/59 | 34/135/170 | 0.373 | 0.299 |
| Portugal | 104 | 190 | 19/46/39 | 19/84/87 | 0.404 | 0.321 |
| UK | 74 | 857 | 13/26/35 | 112/380/365 | 0.351 | 0.352 |
| Netherlands | 159 | 585 | 18/70/71 | 58/283/244 | 0.333 | 0.341 |
| Italy | 44 | 248 | 5/17/22 | 33/116/99 | 0.307 | 0.367 |
| Australia | 162 | 795 | 18/69/75 | 85/362/348 | 0.324 | 0.335 |
| USA | 77 | 300 | 10/37/30 | 32/144/124 | 0.370 | 0.347 |
| **Total replication** | **826** | **3,568** | **108/364/354** | **394/1,615/1,559** | **0.351** | **0.337** |
| **Combined** |  |  |  |  |  |  |
| Austria | 213 | 585 | 21/104/88 | 50/236/299 | 0.343 | 0.287 |
| Belgium | 67 | 285 | 11/33/23 | 31/118/136 | 0.410 | 0.316 |
| USA | 148 | 905 | 23/68/57 | 94/404/407 | 0.385 | 0.327 |
| Finland | 116 | 746 | 9/57/50 | 72/288/386 | 0.323 | 0.290 |
| Ireland | 148 | 209 | 20/78/50 | 28/92/89 | 0.399 | 0.354 |
| UK | 351 | 5,969 | 68/139/144 | 660/2,736/2,573 | 0.392 | 0.340 |
| Switzerland | 182 | 259 | 22/90/70 | 24/113/122 | 0.368 | 0.311 |
| Germany | 150 | 339 | 21/70/59 | 34/135/170 | 0.373 | 0.299 |
| Netherlands | 159 | 585 | 18/70/71 | 58/283/244 | 0.333 | 0.341 |
| Portugal | 104 | 190 | 19/46/39 | 19/84/87 | 0.404 | 0.321 |
| Italy | 44 | 248 | 5/17/22 | 33/116/99 | 0.307 | 0.367 |
| Australia | 162 | 795 | 18/69/75 | 85/362/348 | 0.324 | 0.335 |
| **Total** | **1,844** | **11,115** | **255/841/748** | **1,188/4,967/4,960** | **0.366** | **0.330** |

**Supplementary Table 10S.** The genotype counts and allele frequencies of rs11692675 SNP in all populations in the MTLEHS+FS association study. MAF = minor allele frequency.

| **Population** | **Number of patients with MTLEHS+FS** | **Number of controls** | **Genotype counts in patients (GG/GA/AA)** | **Genotype counts in controls (GG/GA/AA)** | **MAF in patients** | **MAF in controls** |
| --- | --- | --- | --- | --- | --- | --- |
| **Discovery** |  |  |  |  |  |  |
| Austria | 45 | 331 | 4/29/12 | 29/125/177 | 0.411 | 0.276 |
| Belgium | 23 | 285 | 5/11/7 | 31/118/136 | 0.457 | 0.316 |
| USA | 23 | 605 | 5/8/10 | 62/260/283 | 0.391 | 0.317 |
| Finland | 18 | 746 | 2/10/6 | 72/288/386 | 0.389 | 0.290 |
| Ireland | 54 | 209 | 5/30/19 | 28/92/89 | 0.370 | 0.354 |
| UK | 117 | 5,112 | 29/45/43 | 548/2,356/2,208 | 0.440 | 0.338 |
| Switzerland | 61 | 259 | 11/30/20 | 24/113/122 | 0.426 | 0.311 |
| **Total discovery** | **341** | **7,547** | **61/163/117** | **794/3,352/3,401** | **0.418** | **0.327** |
| **Replication** |  |  |  |  |  |  |
| Austria | 18 | 254 | 1/11/6 | 21/111/122 | 0.361 | 0.301 |
| Germany | 72 | 339 | 10/34/28 | 34/135/170 | 0.375 | 0.299 |
| Portugal | 54 | 190 | 12/20/22 | 19/84/87 | 0.407 | 0.321 |
| UK | 38 | 857 | 7/14/17 | 112/380/365 | 0.368 | 0.352 |
| Netherlands | 73 | 585 | 10/31/32 | 58/283/244 | 0.349 | 0.341 |
| Italy | 18 | 248 | 2/5/11 | 33/116/99 | 0.250 | 0.367 |
| Australia | 83 | 795 | 12/32/39 | 85/362/348 | 0.337 | 0.335 |
| USA | 15 | 300 | 2/9/4 | 32/144/124 | 0.433 | 0.347 |
| **Total replication** | **371** | **3,568** | **56/156/159** | **394/1,615/1,559** | **0.361** | **0.337** |
| **Combined** |  |  |  |  |  |  |
| Austria | 63 | 585 | 5/40/18 | 50/236/299 | 0.397 | 0.287 |
| Belgium | 23 | 285 | 5/11/7 | 31/118/136 | 0.457 | 0.316 |
| USA | 38 | 905 | 7/17/14 | 94/404/407 | 0.408 | 0.327 |
| Finland | 18 | 746 | 2/10/6 | 72/288/386 | 0.389 | 0.290 |
| Ireland | 54 | 209 | 5/30/19 | 28/92/89 | 0.370 | 0.354 |
| UK | 155 | 5969 | 36/59/60 | 660/2,736/2,573 | 0.423 | 0.340 |
| Switzerland | 61 | 259 | 11/30/20 | 24/113/122 | 0.426 | 0.311 |
| Germany | 72 | 339 | 10/34/28 | 34/135/170 | 0.375 | 0.299 |
| Netherlands | 73 | 585 | 10/31/32 | 58/283/244 | 0.349 | 0.341 |
| Portugal | 54 | 190 | 12/20/22 | 19/84/87 | 0.407 | 0.321 |
| Italy | 18 | 248 | 2/5/11 | 33/116/99 | 0.250 | 0.367 |
| Australia | 83 | 795 | 12/32/39 | 85/362/348 | 0.337 | 0.335 |
| **Total** | **712** | **11,115** | **117/319/276** | **1,188/4,967/4,960** | **0.388** | **0.330** |

**Rare variants in *SCN1A***

We detected association with common variants, but we cannot completely exclude the possibility of rare variants with large effects creating “synthetic” associations (Dickso*n et al*., 2010). However, there is little evidence for such rare variants of large effect size in *SCN1A* and MTLEHS. MTLEHS very rarely shows familial clustering, to be expected for a disease caused by large-effect variants. Although *SCN1A* is well-studied in familial epilepsies, to our knowledge there have been only two reports of families in which a patient with *SCN1A* mutation had MTLEHS (Abou-Khali*l et al*., 2001; Mantegazz*a et al*., 2005), despite several hundred *SCN1A* mutations described in databases (Clae*s et al*., 2009; Loss*i*n, 2009). We performed exome sequencing in a few patients with a strong family history of epilepsy and/or febrile seizures (at least two affected relatives) and have not detected *SCN1A* coding mutations in rs7587026 risk-allele carriers (data not shown). MTLEHS with familial clustering is rare and we only had 11 patients in this group (two homozygotes and nine heterozygotes for the risk allele).

**References**

Abou-Khalil B, Ge Q, Desai R, Ryther R, Bazyk A, Bailey R, et al. Partial and generalized epilepsy with febrile seizures plus and a novel SCN1A mutation. Neurology 2001; 57: 2265–2272.

Beach TG, Sue LI, Walker DG, Roher AE, Lue L, Vedders L, et al. The Sun Health Research Institute Brain Donation Program: description and experience, 1987-2007. Cell Tissue Bank 2008; 9: 229–245.

Boyd A, Golding J, Macleod J, Lawlor DA, Fraser A, Henderson J, et al. Cohort Profile: The ‘Children of the 90s’--the index offspring of the Avon Longitudinal Study of Parents and Children. International journal of epidemiology 2012

Chomczynski P, Sacchi N. Single-step method of RNA isolation by acid guanidinium thiocyanate-phenol-chloroform extraction. Anal. Biochem. 1987; 162: 156–159.

Cirulli ET, Kasperaviciūte D, Attix DK, Need AC, Ge D, Gibson G, et al. Common genetic variation and performance on standardized cognitive tests. Eur. J. Hum. Genet. 2010; 18: 815–820.

Claes LRF, Deprez L, Suls A, Baets J, Smets K, Van Dyck T, et al. The SCN1A variant database: a novel research and diagnostic tool. Hum. Mutat. 2009; 30: E904–920.

Cronin S, Berger S, Ding J, Schymick JC, Washecka N, Hernandez DG, et al. A genome-wide association study of sporadic ALS in a homogenous Irish population. Hum. Mol. Genet. 2008; 17: 768–774.

Dickson SP, Wang K, Krantz I, Hakonarson H, Goldstein DB. Rare variants create synthetic genome-wide associations. PLoS Biol. 2010; 8: e1000294.

Ghoussaini M, Fletcher O, Michailidou K, Turnbull C, Schmidt MK, Dicks E, et al. Genome-wide association analysis identifies three new breast cancer susceptibility loci. Nat. Genet. 2012; 44: 312–318.

Heinzen EL, Yoon W, Tate SK, Sen A, Wood NW, Sisodiya SM, et al. Nova2 interacts with a cis-acting polymorphism to influence the proportions of drug-responsive splice variants of SCN1A. Am. J. Hum. Genet. 2007; 80: 876–883.

Van der Hel WS, Notenboom RGE, Bos IWM, van Rijen PC, van Veelen CWM, de Graan PNE. Reduced glutamine synthetase in hippocampal areas with neuron loss in temporal lobe epilepsy. Neurology 2005; 64: 326–333.

Howie B, Fuchsberger C, Stephens M, Marchini J, Abecasis GR. Fast and accurate genotype imputation in genome-wide association studies through pre-phasing. Nat. Genet. 2012; 44: 955–959.

International Parkinson’s Disease Genomics Consortium, Wellcome Trust Case Control Consortium 2. A two-stage meta-analysis identifies several new loci for Parkinson’s disease. PLoS Genet. 2011; 7: e1002142.

Irizarry RA, Hobbs B, Collin F, Beazer-Barclay YD, Antonellis KJ, Scherf U, et al. Exploration, normalization, and summaries of high density oligonucleotide array probe level data. Biostatistics 2003; 4: 249–264.

Kasperaviciūte D, Catarino CB, Heinzen EL, Depondt C, Cavalleri GL, Caboclo LO, et al. Common genetic variation and susceptibility to partial epilepsies: a genome-wide association study. Brain 2010; 133: 2136–2147.

Li Y, Willer C, Sanna S, Abecasis G. Genotype imputation. Annu Rev Genomics Hum Genet 2009; 10: 387–406.

Li Y, Willer CJ, Ding J, Scheet P, Abecasis GR. MaCH: using sequence and genotype data to estimate haplotypes and unobserved genotypes. Genet. Epidemiol. 2010; 34: 816–834.

Libioulle C, Louis E, Hansoul S, Sandor C, Farnir F, Franchimont D, et al. Novel Crohn disease locus identified by genome-wide association maps to a gene desert on 5p13.1 and modulates expression of PTGER4. PLoS Genet. 2007; 3: e58.

Livak KJ, Schmittgen TD. Analysis of relative gene expression data using real-time quantitative PCR and the 2(-Delta Delta C(T)) Method. Methods 2001; 25: 402–408.

Lohoff FW, Ferraro TN, Dahl JP, Hildebrandt MA, Scattergood TM, O’Connor MJ, et al. Lack of association between variations in the brain-derived neurotrophic factor (BDNF) gene and temporal lobe epilepsy. Epilepsy Res. 2005; 66: 59–62.

Lossin C. A catalog of SCN1A variants. Brain Dev. 2009; 31: 114–130.

Mägi R, Morris AP. GWAMA: software for genome-wide association meta-analysis. BMC Bioinformatics 2010; 11: 288.

Manna I, Gambardella A, Bianchi A, Striano P, Tozzi R, Aguglia U, et al. A functional polymorphism in the SCN1A gene does not influence antiepileptic drug responsiveness in Italian patients with focal epilepsy. Epilepsia 2011; 52: e40–44.

Mantegazza M, Gambardella A, Rusconi R, Schiavon E, Annesi F, Cassulini RR, et al. Identification of an Nav1.1 sodium channel (SCN1A) loss-of-function mutation associated with familial simple febrile seizures. Proc. Natl. Acad. Sci. U.S.A. 2005; 102: 18177–18182.

Martin MS, Tang B, Ta N, Escayg A. Characterization of 5’ untranslated regions of the voltage-gated sodium channels SCN1A, SCN2A, and SCN3A and identification of cis-conserved noncoding sequences. Genomics 2007; 90: 225–235.

Millar T, Walker R, Arango J-C, Ironside JW, Harrison DJ, MacIntyre DJ, et al. Tissue and organ donation for research in forensic pathology: the MRC Sudden Death Brain and Tissue Bank. J. Pathol. 2007; 213: 369–375.

Moffatt MF, Gut IG, Demenais F, Strachan DP, Bouzigon E, Heath S, et al. A large-scale, consortium-based genomewide association study of asthma. N. Engl. J. Med. 2010; 363: 1211–1221.

Monsuur AJ, de Bakker PIW, Alizadeh BZ, Zhernakova A, Bevova MR, Strengman E, et al. Myosin IXB variant increases the risk of celiac disease and points toward a primary intestinal barrier defect. Nat. Genet. 2005; 37: 1341–1344.

Myllykangas L, Wavrant-De Vrièze F, Polvikoski T, Notkola I-L, Sulkava R, Niinistö L, et al. Chromosome 21 BACE2 haplotype associates with Alzheimer’s disease: a two-stage study. J. Neurol. Sci. 2005; 236: 17–24.

Nalls MA, Plagnol V, Hernandez DG, Sharma M, Sheerin U-M, Saad M, et al. Imputation of sequence variants for identification of genetic risks for Parkinson’s disease: a meta-analysis of genome-wide association studies. Lancet 2011; 377: 641–649.

Need AC, Attix DK, McEvoy JM, Cirulli ET, Linney KL, Hunt P, et al. A genome-wide study of common SNPs and CNVs in cognitive performance in the CANTAB. Hum. Mol. Genet. 2009; 18: 4650–4661.

Patterson N, Price AL, Reich D. Population structure and eigenanalysis. PLoS Genet 2006; 2: e190.

Peuralinna T, Oinas M, Polvikoski T, Paetau A, Sulkava R, Niinistö L, et al. Neurofibrillary tau pathology modulated by genetic variation of alpha-synuclein. Ann. Neurol. 2008; 64: 348–352.

Price AL, Patterson NJ, Plenge RM, Weinblatt ME, Shadick NA, Reich D. Principal components analysis corrects for stratification in genome-wide association studies. Nat. Genet. 2006; 38: 904–909.

Purcell S, Neale B, Todd-Brown K, Thomas L, Ferreira MAR, Bender D, et al. PLINK: a tool set for whole-genome association and population-based linkage analyses. Am. J. Hum. Genet. 2007; 81: 559–575.

Sadleir LG, Scheffer IE. Febrile seizures. BMJ 2007; 334: 307–311.

Schlachter K, Gruber-Sedlmayr U, Stogmann E, Lausecker M, Hotzy C, Balzar J, et al. A splice site variant in the sodium channel gene SCN1A confers risk of febrile seizures. Neurology 2009; 72: 974–978.

Shabalin AA. Matrix eQTL: ultra fast eQTL analysis via large matrix operations. Bioinformatics 2012; 28: 1353–1358.

Silva AM, Bettencourt A, Pereira C, Santos E, Carvalho C, Mendonça D, et al. Protective role of the HLA-A*02 allele in Portuguese patients with multiple sclerosis. Mult. Scler. 2009; 15: 771–774.

Tate SK, Depondt C, Sisodiya SM, Cavalleri GL, Schorge S, Soranzo N, et al. Genetic predictors of the maximum doses patients receive during clinical use of the anti-epileptic drugs carbamazepine and phenytoin. Proc. Natl. Acad. Sci. U.S.A 2005; 102: 5507–5512.

Todt U, Freudenberg J, Goebel I, Heinze A, Heinze-Kuhn K, Rietschel M, et al. Variation of the serotonin transporter gene SLC6A4 in the susceptibility to migraine with aura. Neurology 2006; 67: 1707–1709.

Trabzuni D, Ryten M, Walker R, Smith C, Imran S, Ramasamy A, et al. Quality control parameters on a large dataset of regionally dissected human control brains for whole genome expression studies. J. Neurochem. 2011; 119: 275–282.

Wellcome Trust Case Control Consortium, Australo-Anglo-American Spondylitis Consortium (TASC), Burton PR, Clayton DG, Cardon LR, Craddock N, et al. Association scan of 14,500 nonsynonymous SNPs in four diseases identifies autoimmunity variants. Nat. Genet. 2007; 39: 1329–1337.
